# Versatile sugar and valerate metabolic pathways in * Paraburkholderia xenovorans* LB400 enable tailored poly(3-hydroxybutyrate-*co*-3-hydroxyvalerate) production

**DOI:** 10.1007/s00253-025-13599-8

**Published:** 2025-10-29

**Authors:** Mario I. Sepúlveda, Viviana Urtuvia, Natalia Álvarez-Santullano, Pamela Villegas, Jacqueline Vásquez-Navarrete, Valentina Saffirio, Alvaro Díaz-Barrera, Myriam González, Jose Gregório C. Gomez, Valentina Méndez, Michael Seeger

**Affiliations:** 1 Laboratorio de Microbiología Molecular y Biotecnología Ambiental, Departamento de Química & Centro de Biotecnología Daniel Alkalay Lowitt, Universidad Técnica Federico Santa María, 1680, 2390123 Valparaíso, Chile; 2 Millenium Nucleus Bioproducts, Genomics and Environmental Microbiology (BioGEM), Avenida España 1680, Valparaíso, 2390123 Chile; 3 Escuela de Ingeniería Bioquímica, Pontificia Universidad Católica de Valparaíso, Valparaíso, Av Chile; 4 Programa Doctorado en Ciencias de Materiales e Ingeniería de Procesos, Universidad Tecnológica Metropolitana, 8940000 Santiago, Chile; 5 Nano-Biotechnology Applied Laboratory, Instituto de Agroindustría, Universidad de La Frontera, Temuco, Chile; 6 Department of Microbiology, Institute of Biomedical Sciences, University de São Paulo, São Paulo, Brazil

**Keywords:** *Paraburkholderia xenovorans*; Poly(3-hydroxybutyrate), Poly(3-hydroxybutyrate*-co-*3-hydroxyvalerate), PHA, Genome, Metabolism

## Abstract

**Abstract:**

Poly(3-hydroxybutyrate) and poly(3-hydroxybutyrate-*co*-3-hydroxyvalerate) polymers are accumulated by diverse prokaryotes. Their distinct monomer compositions enable their use as tailored bioplastics. The aims were to characterize the poly(3-hydroxybutyrate) and poly(3-hydroxybutyrate-*co*-3-hydroxyvalerate) synthesis by *Paraburkholderia xenovorans* LB400 using different sugars and valerate, and to gain genome-oriented insights into polyhydroxyalkanoate production. d-Glucose, d-mannitol, d-gluconate, and d-xylose were evaluated as sole carbon sources or supplemented with valerate. Polyhydroxyalkanoates synthesized by strain LB400 were characterized through GC–MS, GC-FID, FTIR, and ^1^H and ^13^C-NMR. *P. xenovorans* LB400 reached 1.00–1.39 g L^−1^ of dry cell weight (DCW) with a P(3HB) content of 21–43% w w^−1^ when grown on different sugars. The addition of valerate to the sugar-grown LB400 cultures yielded a DCW of 1.79 to 2.29 g L^−1^ and a P(3HB-*co*-3HV) content of 50.0‒51.2% w w^−1^, with varying 3HV compositions (28‒43 mol%). The highest 3HV incorporation was observed with d-xylose and valerate. Genomic analyses of strain LB400 revealed key elements of sugar metabolism influencing growth, polymer accumulation, and monomer composition. LB400 genome encodes the PhaJ-like *R*-specific hydratase and FadJ epimerase, which are potentially useful for modulating copolymer composition. PHA production under bioreactor conditions was evaluated. In a bioreactor fed with d-glucose, LB400 achieved a P(3HB) concentration of 2.2 g L^−1^. These findings highlight the metabolic versatility of *P. xenovorans* LB400 in utilizing diverse sugars to produce either P(3HB) or tailor-made P(3HB*-co*-3HV)*,* supporting the development of bioplastics for specific applications.

**Key points:**

• *Strain LB400 produced P(3HB-co-3HV) from various sugars and valerate.*

• *Sugar type drives LB400 PHA copolymer synthesis and composition.*

• *Strain LB400 PHA production was scaled up to a bioreactor.*

**Supplementary Information:**

The online version contains supplementary material available at 10.1007/s00253-025-13599-8.

## Introduction

Polyhydroxyalkanoates (PHAs) are diverse polymers that occur in nature and possess attractive physicochemical properties and advantages as bioplastics for biotechnological applications and sustainable development (Seeger et al. 2025). Properties include thermomechanical versatility, biodegradability, biocompatibility, and biobased source and polymerization, which engage global environmental issues such as plastic pollution and greenhouse gas emissions (Madison & Huisman 1999; Chen 2009).

Biologically synthesized PHAs are hydroxy acid polyesters that accumulate as intracellular granules by diverse prokaryotes, playing a key role in the survival of these microorganisms under unbalanced nutritional conditions and other stresses (e.g., carbon excess and non-carbon nutrient scarcity) (Peña et al. 2014; Obruca et al. 2020). A wide variety of monomers have been described as constituents of PHAs, which are synthesized through diverse metabolic pathways from various carbon sources (Nomura & Taguchi 2007). PHAs containing monomers with 3−5 and 6−14 carbon atoms are classified as short-chain-length (PHA_scl_) and medium-chain-length (PHA_mcl_), respectively (Khanna & Srivastava 2005). The most studied PHA_scl_ homopolymer is poly(3-hydroxybutyrate) (P(3HB)) (Koller & Mukherjee 2022). However, the high melting temperature, brittleness, and stiffness of P(3HB) may limit its applications, whereas PHA_scl_ copolymers, such as poly(3-hydroxybutyrate-*co-*3-hydroxyvalerate) (P(3HB-*co-*3HV)), are less brittle and more flexible than P(3HB) (Reddy et al. 2003). The diversity of PHA copolymer compositions allows their usage in diverse applications, including medicine (e.g., tissue engineering and drug encapsulation devices) (Philip et al. 2007; Gigante et al. 2021; Sanhueza et al. 2021; Vilchez et al. 2021), agriculture (e.g., carriers for agricultural products, mulch films, nets), and food packaging (Kourmentza et al. 2021).

Advances in genomics and PHA metabolism have unveiled the synthetic pathways of 3-hydroxybutyrate (3HB) and 3-hydroxyvalerate (3HV) monomers. This knowledge has enabled the customization of P(3HB-*co-*3HV) copolymer composition to tailor its thermomechanical properties for specific applications (Jung et al. 2019). For instance, increasing the 3HV content by 65% results in a reduction of the melting temperature by 60 °C and glass transition temperature by 3.5°C, along with a 10% decrease in the elongation at the break. These thermomechanical changes enhance processability and reduce polymer brittleness (Volova et al. 2021).

The 3HB monomer is commonly synthesized from acetyl-CoA by the 3-ketothiolase (PhaA) and the acetoacetyl-CoA reductase (PhaB), yielding (*R*)-3-hydroxybutyryl-CoA (*R-*3HB-CoA), which is then polymerized through the PHA synthase (PhaC) enzyme (Steinbüchel 2001). Acetyl-CoA is obtained from the metabolism of sugars that are oxidized through distinct pathways to produce, at different extents, biomass, reduced electron shuttle molecules (e.g., NADPH), and the P(3HB) precursor *R-*3HB-CoA (Álvarez-Santullano et al. 2021). For 3HV monomer synthesis, the related carbon sources, valerate, levulinate, or propionate, have been frequently used (Chang et al. 2018). While the choice of fatty acids has been shown to influence the monomer composition of P(3HB-*co-*3HV), the impact of different sugars on monomer composition has been scarcely studied (Kim et al. 2009). Accordingly, the *R*-specific enoyl-CoA hydratase (PhaJ, MaoC), 3-ketothiolase (BktB), and the (*S*)−3-hydroxyacyl-CoA epimerase (FadB, FadJ) enzymes have been studied in recombinant and model wild-type bacteria for synthesizing customized PHA copolymers from fatty acids and non-related carbon sources (Srirangan et al. 2016; Tan et al. 2022).

Genome-wide analyses allowed the identification of enzymes with unique characteristics in *Paraburkholderia* species. The PhaJ-like enzymes have been identified in this genus through genome-based analyses and may participate in the synthesis of (*R*)-3-hydroxyacyl-CoA from intermediates of fatty acid *β*-oxidation (Álvarez-Santullano et al. 2021). Notably, these enzymes exhibit an acetyl/butyryl phosphotransferase (PTA-PTB) domain that may connect PHA synthesis to acetate metabolism (Wolfe et al., 2005; Álvarez-Santullano et al. 2021). The (*S*)-3-hydroxyacyl-CoA epimerase may divert intermediates of fatty acid *β*-oxidation into PHA precursors, such as *R-*3HB-CoA (Volodina & Steinbüchel 2014; Álvarez-Santullano et al. 2021). These enzymes identified in *Paraburkholderia* species are useful tools for the design and development of PHAs.

To address the challenges associated with PHA production, such as enhancing productivity, customizing monomer composition, scaling up processes, and minimization of costs, the biotechnology industry has developed model wild-type and recombinant strains that utilize pure sugars, such as d-glucose or d-gluconate (Volodina et al. 2016; Tan et al. 2021). However, factors such as mass transfer limitation, agitation, and shear stress present significant restrictions during the scale-up of bioplastic production. These limitations continue to interfere with the broader commercialization of tailored PHA synthesis (Koller & Mukherjee 2022; Thiele et al. 2024). Next-generation industrial biotechnology (NGIB) moves towards the utilization of non-conventional, low-cost substrates, which are composed of a variety of sugars that currently represent up to 50% of PHA production costs when used as pure sugars (da Cruz-Pradella 2021; Vilchez et al. 2025). For example, d-mannitol and d-xylose are highly abundant sugars in algal and lignocellulosic biomass, respectively, highlighting these compounds as relevant carbon sources for PHA production within the NGIB approach (Cesário et al. 2018). Therefore, the search for microorganisms capable of utilizing diverse sugars and enduring various environmental stresses is becoming increasingly necessary for the competitive production of bioplastics intended for biotechnological applications.

Environmental bacteria exhibit diverse metabolic capabilities that enable the production of PHA copolymers with tailored monomer compositions and other biotechnological applications (Fuentes et al. 2014; Álvarez-Santullano et al. 2021). Moreover, their stress-resistance mechanisms offer valuable advantages to overcome challenges associated with scaling-up processes. The model bacterium *Paraburkholderia xenovorans* LB400 possesses a highly versatile metabolism, capable of degrading a wide range of aromatic compounds, including polychlorobiphenyls (PCBs). Additionally, it harbors an extensive array of stress resistance mechanisms (Seeger et al. 1999; Agulló et al. 2007, 2017; Chain et al. 2006; Méndez et al. 2011, 2025; Romero-Silva et al. 2013; Chirino et al. 2013; Vargas-Straube et al. 2016; Urtuvia et al. 2018). A large multi-replicon genome (9.7 Mbp) confers a versatile metabolism to strain LB400 for growth on diverse carbon sources and to synthesize molecules related to nutrient scarcity, such as P(3HB) and a non-ribosomal siderophore (Chain et al. 2006; Vargas-Straube et al. 2016; Urtuvia et al. 2018). A genome-guided metabolic reconstruction of *Paraburkholderia* species, including strain LB400, revealed a highly redundant and robust capacity for sugar and fatty acid catabolism to support growth under harsh environmental conditions, which is an advantageous trait for the biotechnological production of PHAs (Álvarez-Santullano et al. 2021). The hierarchical utilization of sugars by *P. xenovorans* LB400 in PHA synthesis influences the physicochemical properties of P(3HB) electrospun microfibers, including molecular weight and crystallinity degree (Acevedo et al. 2018; Sanhueza et al. 2020). These findings and the metabolic versatility of *P. xenovorans* LB400 enable the NGIB development towards the synthesis of PHAs for their use in added-value applications, such as tissue engineering devices (Álvarez-Santullano et al. 2020).

This study focused on synthesizing the P(3HB) homopolymer and the P(3HB-*co-*3HV) copolymer using *P. xenovorans* LB400 cultivated on various biotechnologically relevant sugars and valerate as carbon and energy sources. The aim was to gain genome-oriented metabolic insight into PHA synthesis and explore its potential applications. Additionally, this study evaluated the scalability of PHA production under bioreactor conditions.

## Materials and methods

### Chemicals

Sugars and valeric acid (purity > 99.9% w w^−1^) were obtained from Sigma-Aldrich (Saint Louis, MO, USA).

### Bacterial strain and growth conditions

*P. xenovorans* LB400 is deposited under the code CCUG 46959, in the Culture Collection University of Gothenburg (CCUG, Gothenburg, Sweden) and in the DSMZ-German Collection of Microorganisms and Cell Cultures (DSMZ, Braunschweig, Germany) under the DSM No. 17367. LB400 cells were grown in LB broth (10 g L^−1^ tryptone, 5 g L^−1^ yeast extract, 5 g L^1^ NaCl) or in M9 minimal medium supplemented with the respective sugar (1.8–10 g L^−1^) in an agitated flask at 150 rpm and 30 °C. One liter of M9 minimal medium was prepared with 6.78 g Na_2_HPO_4_, 3 g KH_2_PO_4_, 0.5 g NaCl, and 1 g NH_4_Cl. Trace solutions consisted of 154 mg MgSO_4_ × 7H_2_O, 13.4 mg MgCl_2_ × 6H_2_O, 11.9 mg FeSO_4_ × 7H_2_O, 2.5 mg CaCO_3_, 1.8 mg ZnSO_4_ × 7H_2_O, 1.4 mg MnSO_4_ × H_2_O, 0.35 mg CoCl_2_ × 6H_2_O, 0.3 mg CuSO_4_ × 5H_2_O, and 0.075 mg H_3_BO_3_ per liter of M9 medium and 0.0024% v v^−1^ HCl (Urtuvia et al. 2018). LB400 cells grown until stationary phase in LB medium were used to inoculate (10% v v^−1^) M9 cultures in flasks or bioreactors. All the experiments were carried out at least in triplicate.

### Assessment of PHA production during bacterial growth

To promote P(3HB) production, LB400 cells were incubated in modified M9 minimal medium (high initial C:N ratio) with 10-fold less ammonium chloride concentration (0.1 g L^−1^ NH_4_Cl), and with 10 g L^−1^ d-mannitol, d-glucose, d-gluconate, or d-xylose for 48 h (Sanhueza et al. 2020). As a control medium, cultures grown in M9 minimal medium with 1.8 g L^−1^ mannitol were included (low initial C:N ratio). High and low C:N ratio media were used to grow strain LB400 until the stationary phase to observe PHA granules through transmission electron microscopy. Cell preparation and transmission electron microscopy were done as described by Nurbas & Kutsal (2004). To evaluate the synthesis of P(3HB-*co-*3HV) copolymers, strain LB400 was grown in flasks (250 mL) with nitrogen-limited M9 medium, and after 24 h, a pulse of 1 g L^−1^ valeric acid neutralized with sodium hydroxide was added, maintaining the culture for further 24 h. PHA cell content and monomer composition were assessed through GC–MS and GC-FID techniques after 48 h of culture.

To assess growth and P(3HB) production kinetics, strain LB400 was grown in agitated flasks of a 50-mL working volume and a bioreactor of 3 L (Applikon Biotechnology, Delft, The Netherlands) with nitrogen-limited M9 medium and 20 g L^−1^ d-glucose during 48 h at 30ºC and 180 rpm. Bioreactor cultures used a working volume of 2 L with controlled pH (pH = 7.0) by the addition of 1.7 M NaOH using an automatic pH controller Alpha pH800 (Thermo Fisher, Waltham, MA, USA). The bioreactor was equipped with two Rushton turbines and agitated at 250 rpm, while air was supplied to the culture at 2 L min^−1^ (1 vvm). Dissolved oxygen was determined by an online polarographic sensor Ingold (Mettler-Toledo, Mexico City, Mexico). d-Glucose was determined through the dinitrosalicylic acid (DNS) method (Miller 1959), while ammonium was quantified using a phenol-hypochlorite colorimetric method (Kaplan 1969).

### Determination of biomass, PHA yields, and growth kinetics

Dry cell weight (DCW) was measured using freeze-dried cells. The biomass to substrate and PHA to substrate yields (*Y*_*x*/*s*_ and *Y*_PHA/*s*_, respectively) were calculated according to the following equations:

1 $$Y_{x/s}=\frac{{X}_{\mathrm{max}} - {X}_{o}}{{S}_{0}-{S}_{t}}$$

2 $$Y_{PHA/s}=\frac{{\mathrm{PHA}}_{\mathrm{max}} - {\mathrm{PHA}}_{o}}{{S}_{0}-{S}_{t}}$$

${X}_{\mathrm{max}}$ is the maximum cell concentration (g L^−1^) during the cell stationary phase, ${X}_{o}$ is the initial cell concentration (g L^−1^), ${P\mathrm{HA}}_{o}$ is the initial PHA concentration (g L^−1^*)*, ${\mathrm{PHA}}_{\mathrm{max}}$ is the maximum PHA concentration (g L^−1^) during the cell stationary phase, ${S}_{t}$ is the sugar concentration (g L^−1^) when the biomass or PHA is maximal, and ${S}_{o}$ is the initial sugar concentration (g L^−1^).

### Physicochemical characterization of the polymers

3-Hydroxybutyrate (3HB) and 3-hydroxyvalerate (3HV) were quantified with an Agilent 7890 A gas chromatography coupled to mass spectrometer (GC–MS) and a GC with flame ionization detection (GC-FID). Samples of 10–20 mg of dried biomass were subjected to propanolysis reaction (Riis & Mai 1988). The GC–MS system was equipped with an HP5 capillary column. Helium (0.8 mL min^−1^) was used as carrier gas with a sample split 1:25. Injector and FID temperatures were 250 and 300 °C, respectively. The oven was programmed at 100 °C for 1 min, increasing temperature at a rate of 8 °C min^−1^ up to 210 °C, which was maintained for 15 min. Benzoic acid was used as an internal standard. External standards were P(3HB) and P(3HB-*co-*3HV) (Sigma-Aldrich, Germany). Monomers were identified by GC–MS. Samples (1 μL) were injected in a splitless mode into a GC–MS system consisting of an Autosystem XL gas chromatograph (Perkin-Elmer, Boston, MA, USA) with an MDN-1 column (Supelco, Bellefonte, PA, USA) coupled to a Turbo Mass spectrometer (Perkin-Elmer, Boston, MA, USA) using helium as a carrier gas.

The obtained PHA polymers were purified with a chloroform extraction, precipitated with chilled methanol, and rinsed with acetone. Samples of P(3HB) and P(3HB-*co-*3HV) polymers (10 mg) were dissolved in deuterated chloroform (CDCL_3_), and ^1^H and ^13^C nuclear magnetic resonance (NMR) spectra were obtained by a Bruker BioSpin spectrometer at 500 MHz (Mendonça et al. 2014; Chang et al. 2018). Infrared spectra for the samples were recorded using a Nicollet model 6700 Fourier transform infrared (FTIR) spectrometer as reported (Nurbas & Kutsal 2004). The spectral values were collected between 4000 and 400 cm^−1^. For sample preparation, the polymer was rolled flat on a KBr chip before analysis.

### Genomic analyses of the PHA metabolic pathways from sugars and valerate in *P. xenovorans* LB400

Metabolic pathways for d-glucose, d-mannitol, d-gluconate, d-xylose, and valerate were reconstructed from genomic data of *P. xenovorans* LB400. Genomic data is available in the RefSeq and the Kyoto Encyclopedia of Genes and Genomes (KEGG) databases (Kanehisa et al. 2022). All enzymes were identified through a bi-directional best-hit approach using the Swiss-Prot database, and sequence identity values higher than 30% with more than 70% of coverage were considered homologs (Wolf et al*.,* 2012). Phylogenetic analyses of the amino acidic sequences of PhaJ and Fad enzymes were carried out through a multiple sequence alignment (MAFFT V.7 software), followed by a phylogenetic tree reconstruction using the Maximum-likelihood algorithm (RaxML V.8 software) with the LG + G model and 1000 bootstrap repetitions (Stamatakis 2016; Katoh & Toh 2008). The tree topology obtained with the maximum likelihood algorithm was corroborated with additional algorithms based on Bayesian inference and minimum evolution and different alignment trimming methods. Genomic contexts were analyzed in the Genome Browser of the KEGG database and the Blast tool (Kanehisa et al. 2022). Enzymes were analyzed using the InterProScan tool of the InterPro database for the prediction of structural domains and protein families (Paysan-Lafosse et al. 2023).

## Results

### *P. xenovorans* LB400 synthesizes P(3HB) from different sugars

*P. xenovorans* LB400 possesses a versatile metabolism for assimilating different carbon sources. d-Glucose, d-mannitol, d-gluconate, or d-xylose were selected as carbon sources to synthesize PHAs due to their biotechnological relevance. The PHA obtained by strain LB400 from different sugars was structurally and physicochemically characterized, while biomass production and polymer content were assessed.

**Fig. 1:**
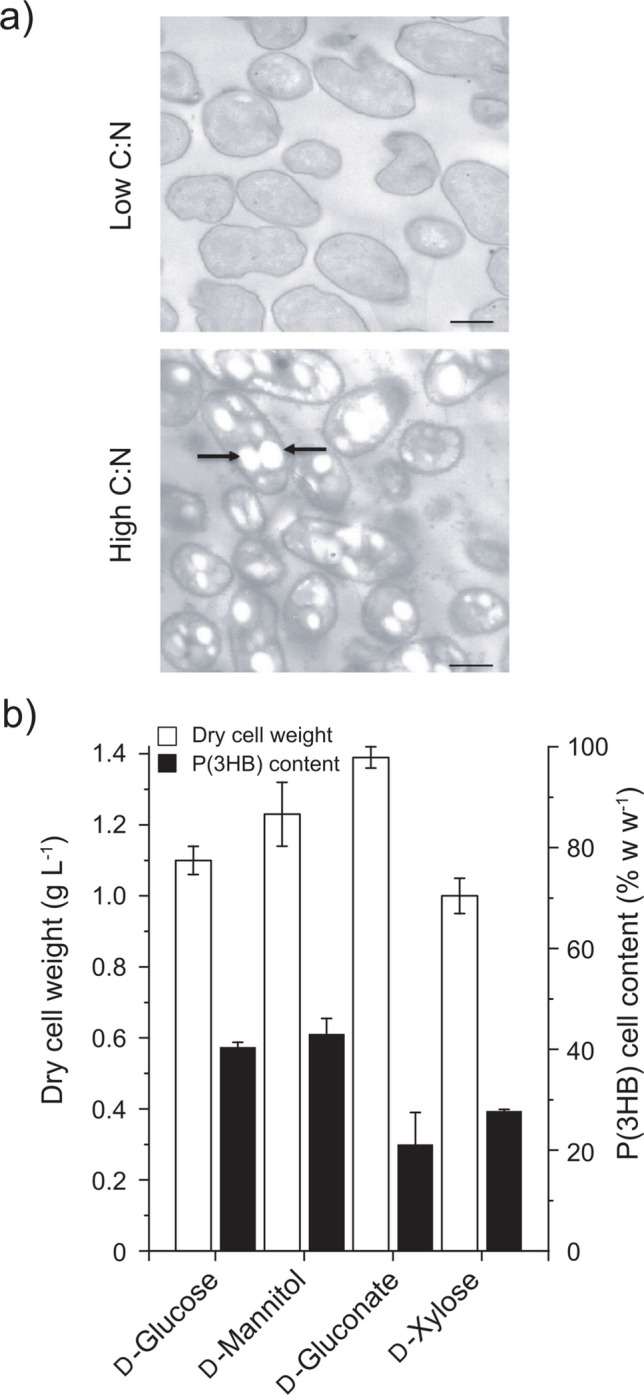
*P. xenovorans* LB400 synthesizes P(3HB) from different sugars. **a**, **b** P(3HB) granules in LB400 cells observed by transmission electron microscopy (12,000 ×). Bacterial cells were grown in control conditions (**a**) with a low C:N ratio (M9 minimal medium using D-mannitol 1.8 g L^−1^ as sole carbon source) and in P(3HB) production medium (**b**) with a high C:N ratio (nitrogen-reduced M9 mineral medium with D-mannitol 10 g L^−^) during 24 h. Arrows indicate P(3HB) granules within the cell. Bar, 1 μm. **c** Biomass and P(3HB) production by strain LB400 using D-glucose, D-mannitol, D-gluconate, and D-xylose (10 g L^−1^) as the sole carbon source

The morphology and intracellular PHA granules in LB400 cells were observed by transmission electron microscopy during growth in a high C:N ratio consisting of an excess d-mannitol and tenfold less ammonium chloride (0.1 g L^−1^). Three to seven granules were observed in the cytoplasm of cells grown on d-mannitol with a high initial C:N ratio (Fig. 1a), reaching a P(3HB) DCW content of 43 ± 3.13% w w^−1^ according to GC–MS determination. In contrast, intracellular PHA granules were absent in cells grown on low C:N ratio conditions (Fig. 1b), consisting of 1 g L^−1^ d-mannitol as the sole carbon source and 1 g L^−1^ of ammonium chloride.

The chemical structure of the P(3HB) produced by *P. xenovorans* LB400 in batch cultures with different selected sugars was characterized. The 3HB propyl esters, obtained after propanolysis of the P(3HB) polymers, showed a retention time of 8.48 min (Figure S1a). Their mass spectra showed the fragment m/z = 131 and m/z = 89, characteristics of all propyl esters of 3-hydroxyacids (Silva-Queiroz et al. 2009). The fragment m/z = 87 corresponds to the [M-CH_3_(CH_2_)_2_O]^+^ fragment characteristic of 3HB propyl ester (Figure S1b). The FTIR spectra of the P(3HB) polymer showed signals associated with the ester-carbonyl functional groups (Table S1).

The NMR spectra of the polymer synthesized from d-glucose, d-mannitol, d-gluconate, and d-xylose by strain LB400 indicated the presence of pure P(3HB). The ^1^H-NMR and ^13^C-NMR shifts of the polymer produced by strain LB400 using d-mannitol are consistent with those associated with the P(3HB) structure (Figure S1). In the ^1^H-NMR spectrum (Figure S1c), the 5.25 and 1.25 ppm signals were characteristic of -CH- and -CH_3_ protons. The signal for the methylene group (−CH_2_) showed a doublet at 2.57 and 2.45 ppm. The ^13^C-NMR spectrum (Figure S1d) showed signals at 169.18, 67.64, 40.82, and 19.79 ppm, assigned to the carbonyl groups −CH-, −CH_2_−, and −CH_3_, respectively.

*P. xenovorans* LB400 presented different biomass and P(3HB) levels when grown with different sugars as the sole carbon and energy sources (Fig. 1c). The cultures grown with d-glucose reached a DCW of 1.10 ± 0.04 g L^−1^ with 40.4 ± 0.9% w w^−1^ of P(3HB) content. The biomass of d-mannitol-grown cells reached 1.23 ± 0.09 g L^−1^ of DCW with an intracellular P(3HB) content of 43.0 ± 3.13% w w^−1^ after 48 h incubation. Using d-gluconate, strain LB400 produced the highest DCW (1.39 ± 0.03 g L^−1^) but the lowest P(3HB) DCW content (21.11 ± 6.37% w w^−1^). The lowest biomass was observed with d-xylose, reaching 1.0 ± 0.05 g L^−1^, with an intracellular P(3HB) DCW content of 27.77 ± 0.29% w w^−1^ (Fig. 1c). All sugars exhibited similar yields for biomass (*Y*_*x/s*_) and P(3HB) (*Y*_PHB/*s*_) synthesis, with values of ~ 0.12 g g^−1^ and ~ 0.03 g g^−1^, respectively. Final pH of all LB400 cultures was 6.7–6.8, except the culture of D-gluconate-fed cells, reaching a final pH of 7.6.

### *P. xenovorans* LB400 synthesizes P(3HB-co-3HV) from different sugars plus valerate

**Fig. 2:**
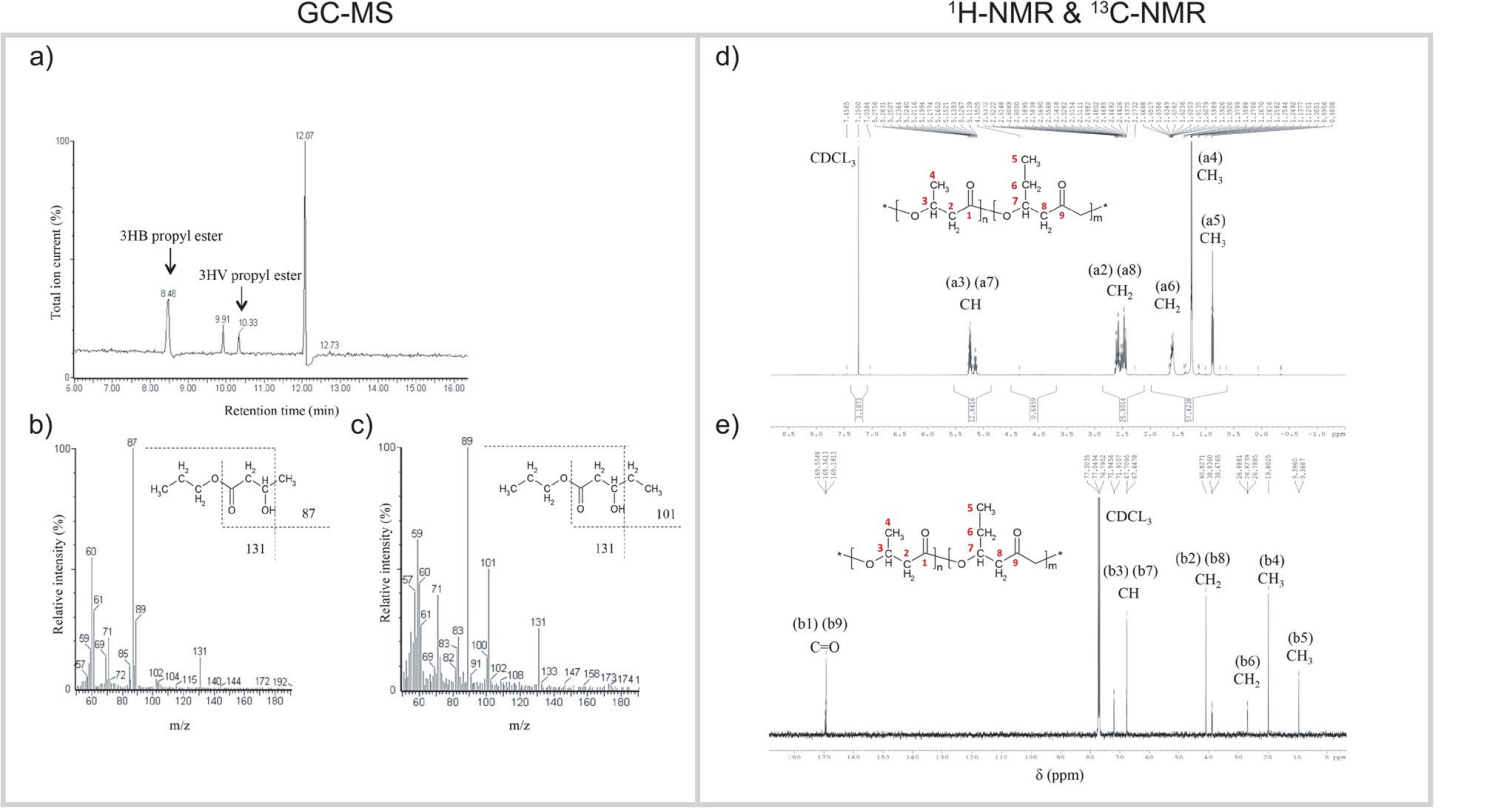
Characterization of P(3HB-*co-*3HV) copolymer synthesized by *P. xenovorans* LB400. **a** Gas chromatography profile of the propyl esters of 3HB and 3HV, with retention times of 8.48 and 10.33 min, respectively. The internal standard benzoate shows a retention time of 12.07 min. **b** The Mass spectrum of 3HB propyl ester shows the characteristic fragment ions [M-CH_3_]^+^ (*m/z* 131) and [M-CH_3_(CH_2_)_2_O]^+^ (*m/z* 87). **c** Mass spectrum of 3HV propyl ester shows the characteristic fragment ions [M-CH_2_CH_3_]^+^ (*m/z* 131) and [M-CH_3_(CH_2_)_2_O]^+^ (*m/z* 101). **d** ^1^H-NMR and **e** ^13^C-NMR spectra obtained from P(3HB-co-3HV) produced from mannitol by strain LB400. The resonance signals were labeled with the appropriate hydrogen and carbon atoms of 3HB and 3HV monomers

*P. xenovorans* LB400 cells were fed with a pulse of valerate during growth using d-glucose, d-mannitol, d-gluconate, or d-xylose to determine the structure of the resultant PHA copolymer and to assess the differences in growth, polymer content, and monomer composition. The propyl esters of 3HB and 3HV obtained after propanolysis of the P(3HB) and P(3HB-*co-*3HV) polymers showed retention times of 8.48 and 10.33 min, respectively (Fig. 2). Both mass spectra showed the m/z = 131 and m/z = 89 fragments characteristic of all propyl esters of 3-hydroxyacids (Silva-Queiroz et al. 2009). The m/z = 87 and m/z = 101 [M-CH_3_(CH_2_)_2_O]^+^ fragments were characteristic of the propyl esters of 3HB and 3HV monomers, respectively, indicating the presence of P(3HB-*co*−3HV) in *P. xenovorans* LB400 cells grown on different sugars and valerate (Nurbas & Kutsal 2004; Mendonça et al. 2014). The FTIR characterization of the P(3HB-*co-*3HV) polymer showed a strong presence of ester bonds and different alkyl groups, confirming that the molecule is a PHA polymer (Table S3).

To further characterize polymer structures synthesized by *P. xenovorans* LB400 from d-glucose, d-mannitol, d-gluconate, or d-xylose with valerate, they were analyzed by ^1^H-NMR and ^13^C-NMR (Fig. 2). The obtained signals in the spectra were similar to those previously described for P(3HB-*co*−3HV) (Keenan et al. 2004; Mendonça et al. 2014; Chang et al. 2018). The ^1^H-NMR spectrum showed signals at 5.25, 1.59, and 1.25 ppm that are characteristic of −CH, −CH_2_− adjacent to CH_3−_ and −CH_3_, respectively (Table S4). In the ^13^C-NMR spectrum, the carbonyl signals at 169.55, 169.36, and 169.18 ppm are associated with 3HB-3HB, 3HB-3HV, and 3HV-3HV, respectively.

**Fig. 3:**
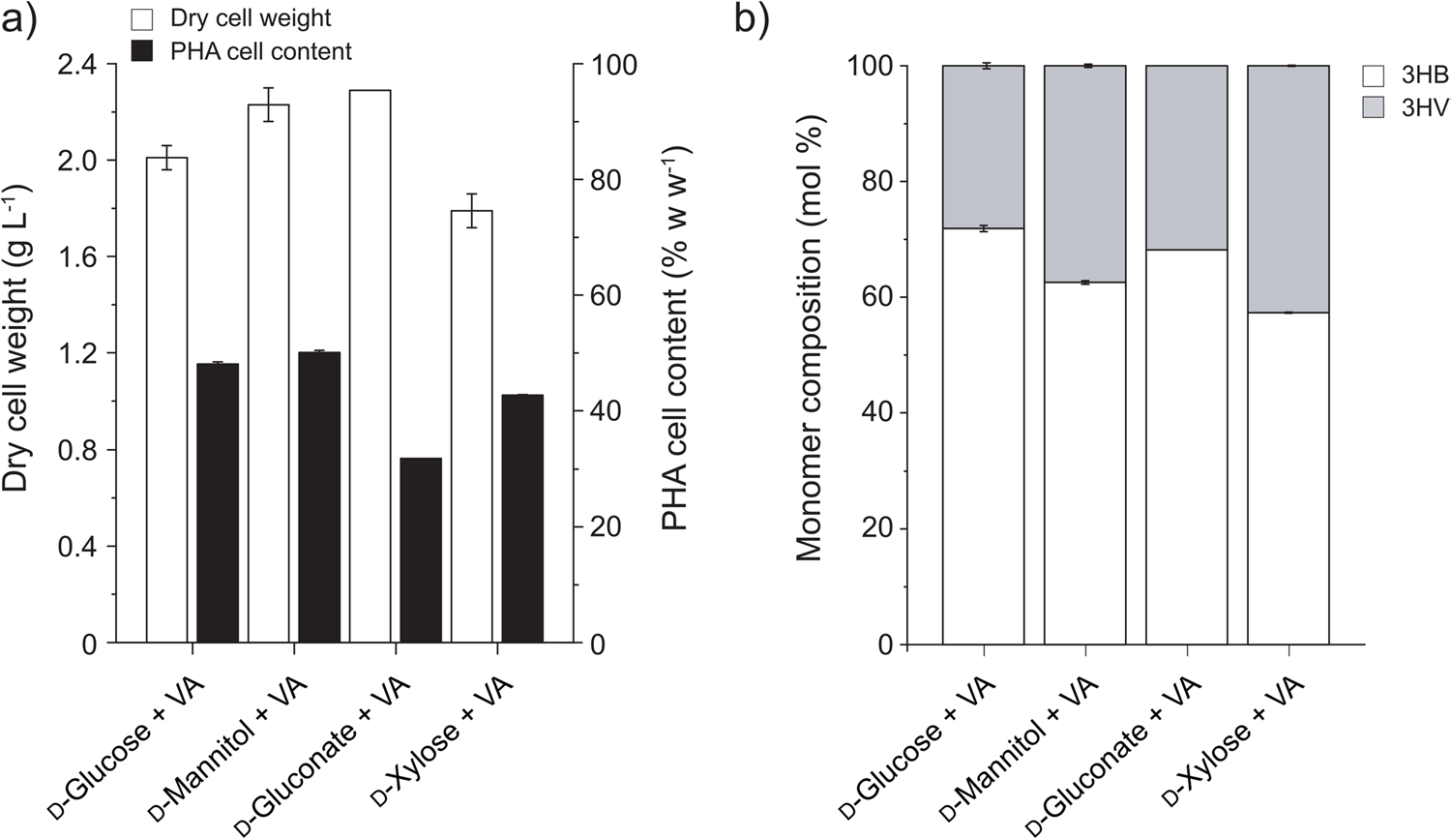
*P. xenovorans* LB400 synthesized P(3HB-*co-*3HV) from different sugars plus valerate (VA). **a** Biomass and PHA content. **b** 3-hydroxybutyrate (3HB) and 3-hydroxyvalerate (3HV) monomer content. Valerate was added after 24 h of incubation

Valerate addition to the LB400 cells grown on different sugars increased the cell growth and PHA content (Fig. 3). Furthermore, sugar selection affected the monomer composition of the P(3HB-*co-*3HV) obtained by *P. xenovorans* LB400 upon valerate addition. The highest LB400 biomass (2.23‒2.29 g L^−1^ DCW) and a P(3HB-*co-*3HV) DCW content (50.05‒51.24% w w^−1^) were observed using d-mannitol or d-gluconate with valerate, respectively. When d-glucose or d-xylose and valerate were supplied, strain LB400 grew up to 2.01 and 1.79 g L^−1^ of DCW, with a P(3HB-*co*−3HV) content of 48.06 and 39.23% w w^−1^, respectively. The highest 3HV incorporation was observed with d-xylose (42.70 ± 0.1 mol%), followed by d-mannitol (37.45 ± 0.30 mol%), d-gluconate (31.83 ± 0.01 mol%), and finally, d-glucose (28.13 ± 0.53 mol%), although d-xylose showed the lowest growth and P(3HB-*co*−3HV) yields (Fig. 3). All cultures grown on different sugars plus valerate exhibited similar yields (*Y*_*x/s*_ ~ 0.20 g g^−1^; *Y*_PHA/*s*_ ~ 0.04 g g^−1^). However, these yields were higher than those of sugar-grown cultures without valerate. Final pH of all LB400 cultures with each sugar plus valerate was 6.6.

To assess the effect of valerate upon growth and PHA accumulation, cultures grown with pure sugars (Fig. 1b) were compared with cultures grown with a sugar and valerate (Fig. 3a). The addition of valerate to *P. xenovorans* LB400 cultures growing on d-glucose, d-mannitol, d-gluconate, or d-xylose incremented the DCW values by 1.13, 0.78, 0.90, and 0.79 g L^−1^, respectively, when compared to a single sugar condition. In cultures grown with d-gluconate plus valerate, PHA content increased by 97.77% of total DCW compared with cultures grown with d-gluconate alone. Otherwise, in cultures with d-glucose, d-mannitol, or d-xylose plus valerate, the PHA content increased in the range of 41.29‒46.26% w w^−1^, corresponding to a PHA concentration increase of 0.42–0.87 g L^−1^ compared to single sugar conditions in all the tested sugars. The increase of PHA concentration in sugar-grown cultures plus valerate compared to those without valerate may be explained by the incorporation of 3HV monomers, representing a PHA concentration increase of 0.29–0.46 g L^−1^, while the remaining PHA concentration increment of 0.40–0.79 g L^−1^ is attributed to an increase of 3HB monomer incorporation.

### Metabolic pathways of *P. xenovorans* LB400 for P(3HB) synthesis from different sugars

**Fig. 4:**
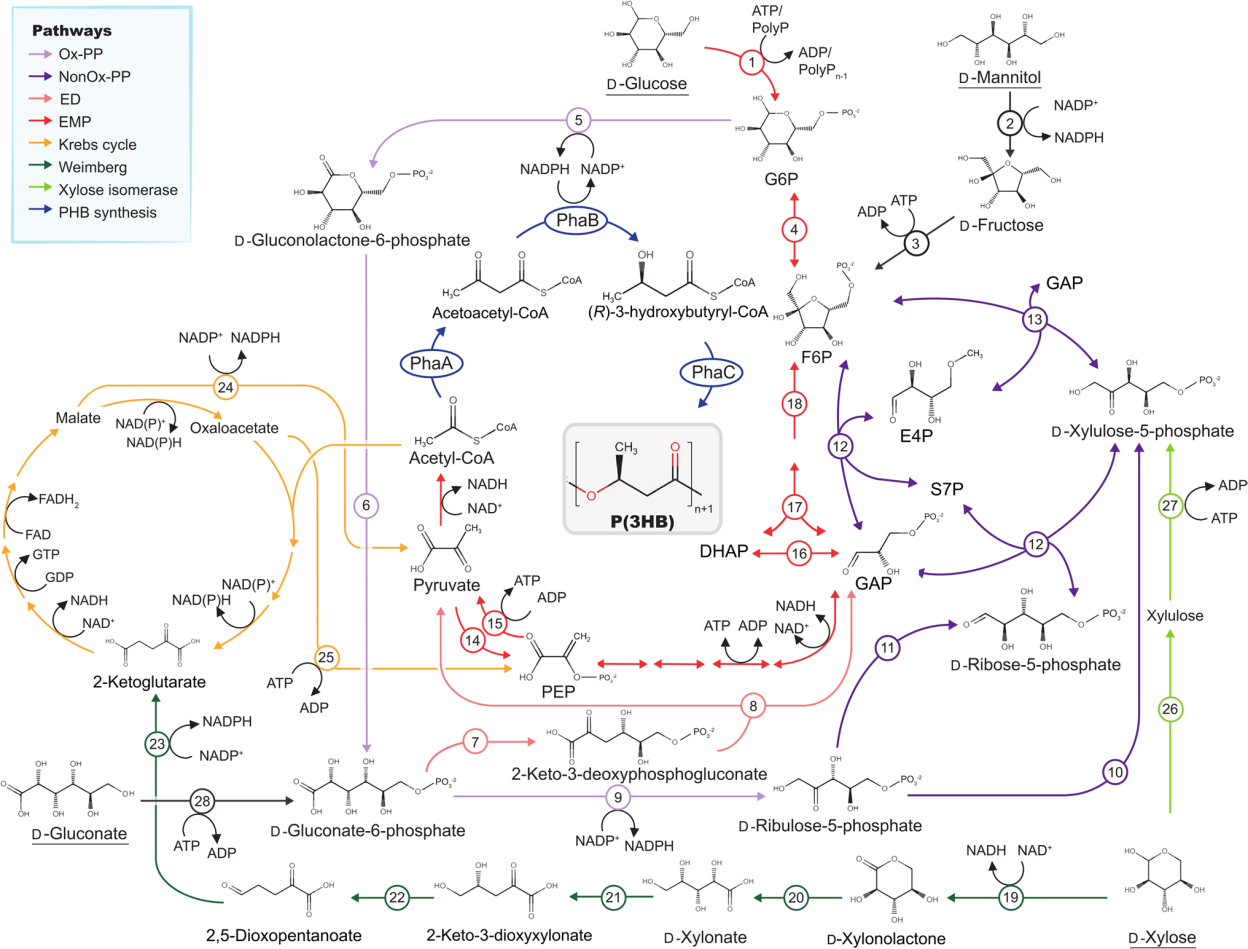
Metabolic pathways of *P. xenovorans* LB400 for the synthesis of P(3HB) from different sugars. 1, ATP-dependent bifunctional glucokinase – HexR transcriptional regulator (Glk) or polyphosphate (polyP) dependent glucokinase (PpgK). 2, mannitol dehydrogenase (MltK). 3, fructokinase (ScrK). 4, fructose-6-phosphate (F6P) isomerase (Pgi). 5, glucose-6-phosphate (G6P) dehydrogenase (Zwf). 6, 6-phosphogluconolactonase (Pgl). 7, gluconate-6-phosphate dehydratase (Edd). 8, 2-keto-3-deoxyphosphogluconate aldolase (Eda). 9, gluconate-6-phosphate dehydrogenase (Pgdh). 10, ribulose-5-phosphate (R5P) isomerase (Rpi). 11, R5P epimerase (Rpe). 12, Transketolase (Tkt). 13, transaldolase (Tal). 14, phosphoenolpyruvate (PEP) synthase (PpsA) or pyruvate dikinase (PpdK). 15, pyruvate kinase (KpyK). 16, triosephosphate isomerase (Tpi). 17, fructose-1,6-bisphosphate (FBP) aldolase (CbbA). 18, FBP phosphatase (Fbp); 19, D-xylose dehydrogenase (XylA). 20, xylonolactonase (XylB). 21, xylonate dehydrogenase (XylC). 22, 2-keto-3-deoxyxylonate dehydratase (XylX). 23, 2,5-dioxopentanoate dehydrogenase (KviD). 24, malic enzyme (MaeB). 25, PEP carboxykinase (PckG); 26, D-xylose isomerase. 27, D-Xylulokinase. 28, gluconate kinase (GntK). OxPP, oxidative branch of the pentose-phosphate pathway (PP). NonOxPP, non-oxidative branch of PP pathway. EMP, Embden-Meyerhof-Parnas. ED, Entner-Doudoroff, Krebs cycle. Metabolic pathways were reconstructed based on the genome of strain LB400. Enzymes are detailed in Table S5

To gain further insights into the differences in biomass formation and synthesis of PHA observed in strain LB400, metabolic pathways of d-glucose, d-mannitol, d-gluconate, and d-xylose (Fig. 4) were reconstructed in silico. Genes and enzymatic functions are detailed in Table S5.

In general, *P. xenovorans* LB400 metabolizes sugars into acetyl-CoA that can be anabolized into the P(3HB) monomer precursor, *R-*3HB-CoA, by the PhaA and PhaB1 (BxeA2341) or PhaB2 (BxeB0354) enzymes. Finally, *R-*3HB-CoA is polymerized by the PHA synthases PhaC1 (BxeA2343), PhaC2 (BxeB0358), and PhaC3 (BxeC0053) (Urtuvia et al. 2018; Álvarez-Santullano et al. 2021).

d-Glucose is imported in *P. xenovorans* LB400 through ATP-binding cassette (ABC) family transporters and subsequently phosphorylated into glucose-6-phosphate (G6P) by the glucokinase enzyme (Glk; BxeA3454) (Daddaoua et al. 2009). *P. xenovorans* LB400 lacks a complete glycolytic Embden-Meyerhof-Parnas (EMP) pathway due to the absence of the phosphofructokinase enzyme, Pfk (Álvarez-Santullano et al. 2021). Therefore, G6P is channeled through the oxidative branch of the pentose-phosphate pathway (OxPP) via G6P dehydrogenase (Zwf), yielding gluconolactone-6-phosphate and NADPH. Strain LB400 harbors three Zwf enzymes encoded by the *zwf* genes: *zwf1* (BxeA3452) gene, the *zwf2* gene (BxeB0215), and the *zwf3* gene (BxeB1764). Gluconolactone-6-phosphate undergoes oxidation by a phosphogluconolactonase (Pgl) into gluconate-6-phosphate (6PG). The enzymes Pgl (BxeA3452), Glk, and Zwf1 are encoded in the *zwf1-pgl-glk* gene cluster and next to ABC hexose transporters. 6PG can be funneled into the ED pathway via 6PG dehydratase (Edd) and 2-keto-3-deoxyphosphogluconate (KDPG) aldolase (Eda), yielding pyruvate and glyceraldehyde-3-phosphate (GAP) (Fig. 4). 6PG may also be obtained from d-gluconate, imported by the gluconate permease (*gntT*; BxeA0592), and subsequently phosphorylated by the gluconokinase (*gntK*; BxeA0591) enzyme. These genes are located next to the *edd* (BxeA0590) and *eda* (BxeA0589) genes in the *eda-edd-gntT-gntK* arrangement.

D-Mannitol in *P. xenovorans* LB400 is likely imported through ABC transporters and oxidized into D-fructose via mannitol dehydrogenase (MltK; BxeA0730), yielding one NADPH equivalent. D-Fructose is phosphorylated into F6P by a fructokinase (ScrK; BxeA4286) and then can be isomerized into G6P by a phosphoglucose isomerase (Pgi; BxeA2287). Otherwise, F6P may enter the Non-OxPP pathway by the transketolase enzyme according to GAP availability obtained through the ED pathway (Pastor et al. 2019).

D-Xylose in *P. xenovorans* LB400 can be catabolized through the Weimberg pathway or the xylose isomerase (XI) pathway (Fig. 4). The Weimberg pathway is encoded in the megaplasmid in an operon-like structure, while the XI pathway is in a distant locus (Table S5). The Weimberg pathway is similar to a non-phosphorylative ED pathway that oxidizes d-xylose through a d-xylose dehydrogenase enzyme, obtaining D-xylonolactone and one NADH equivalent (Tai et al. 2016). d-Xylonolactone is oxidized by xylonolactonase and a xylonate dehydratase into 2-keto-3-deoxyxylonate (KDX), which is then funneled into the Krebs cycle via 2-ketoglutarate, obtained by the dehydration and subsequent oxidation of KDX (Fig. 4). Strain LB400 possesses several enzymes that convert Krebs cycle intermediates into the precursor of P(3HB), acetyl-CoA, including the malic enzymes (MaeB) and phosphoenolpyruvate carboxykinase (PckG) (Stephens et al. 2007). The xylose isomerase pathway metabolizes d-xylose into the Non-OxPP pathway via d-xylulose-5-phosphate (Fig. 4) (Guamán et al. 2018)*.*

### Metabolic pathways for P(3HB-co-3HV) synthesis from different sugars and valerate

**Fig. 5:**
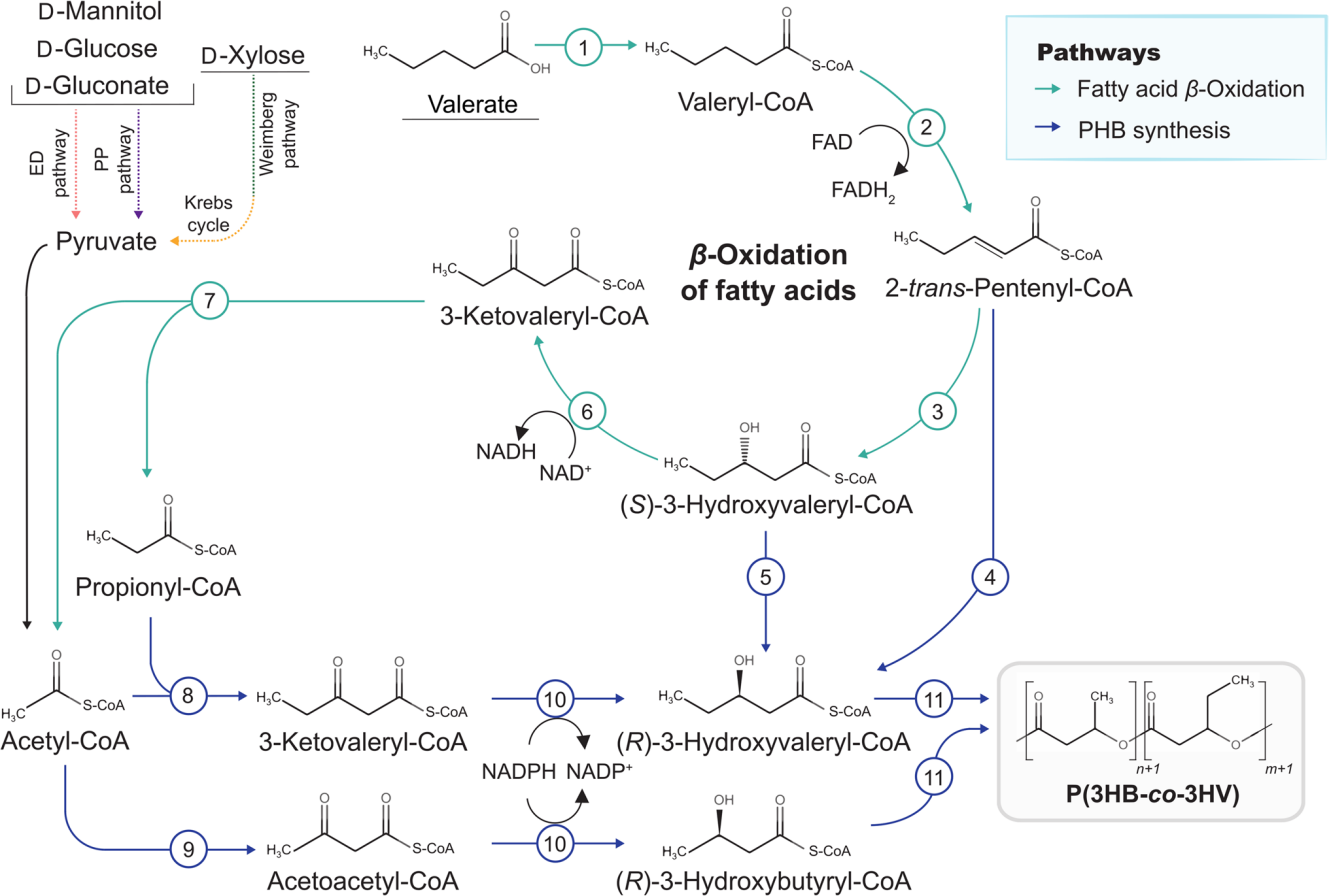
Metabolic pathways of *P. xenovorans* LB400 for the synthesis of P(3HB-*co-*3HV) copolymer from different sugars and valerate. 1, fatty acid-CoA ligase (FadD). 2, acyl-CoA dehydrogenase (FadE, Acad). 3, *S*-specific enoyl-CoA hydratase (ECH) (FadJ, FadN or FadB). 4, *R*-specific enoyl-CoA hydratase (PhaJ). 5, (*S*)−3-hydroxyacyl-CoA (*S-*3HA-CoA) epimerase (FadJ). 6, *S*−3HA-CoA dehydrogenase (FadJ, FadN or FadB). 7, 3-ketoacyl-CoA thiolase (FadI or FadA). 8, 3-ketothiolase (BktB). 9, 3-ketothiolase (PhaA). 10, 3-ketoacyl-CoA reductase (PhaB or FabG). 11, polyhydroxyalkanoate synthase (PhaC). ED, Entner-Doudoroff. Metabolic pathways are reconstructed based on the genome of strain LB400. Enzymes are detailed in Table S6

The pathways of *P. xenovorans* LB400 for the synthesis of 3HV and 3HB monomers were assessed to gain metabolic insights on the relationship between the sugar used as substrate and monomer composition of P(3HB-*co-*3HV) (Fig. 5). In strain LB400, valerate is activated and then undergoes *β*-oxidation, yielding the intermediates 2-*trans*-pentenyl-CoA, (*S*)-3-hydroxyvaleryl-CoA (*S-*3HV-CoA), 3-ketovaleryl-CoA, propionyl-CoA, and acetyl-CoA that can be converted into the 3HV monomer precursor, *R-*3HV-CoA.

The 2-*trans*-pentenyl-CoA intermediate can be hydrated by an *R*-specific enoyl-CoA hydratase (PhaJ) into *R*−3HV-CoA (Tsuge et al. 2000). Strain LB400 harbors two PhaJ-like enzymes with an *R*-specific hydratase (*R*-ECH) domain (previously described as a MaoC-like domain) (Teufel et al. 2010) at the C-terminal, while the N-terminal exhibits a phosphate acetyl-butyryl transferase domain (PTA-PTB).

**Fig. 6:**
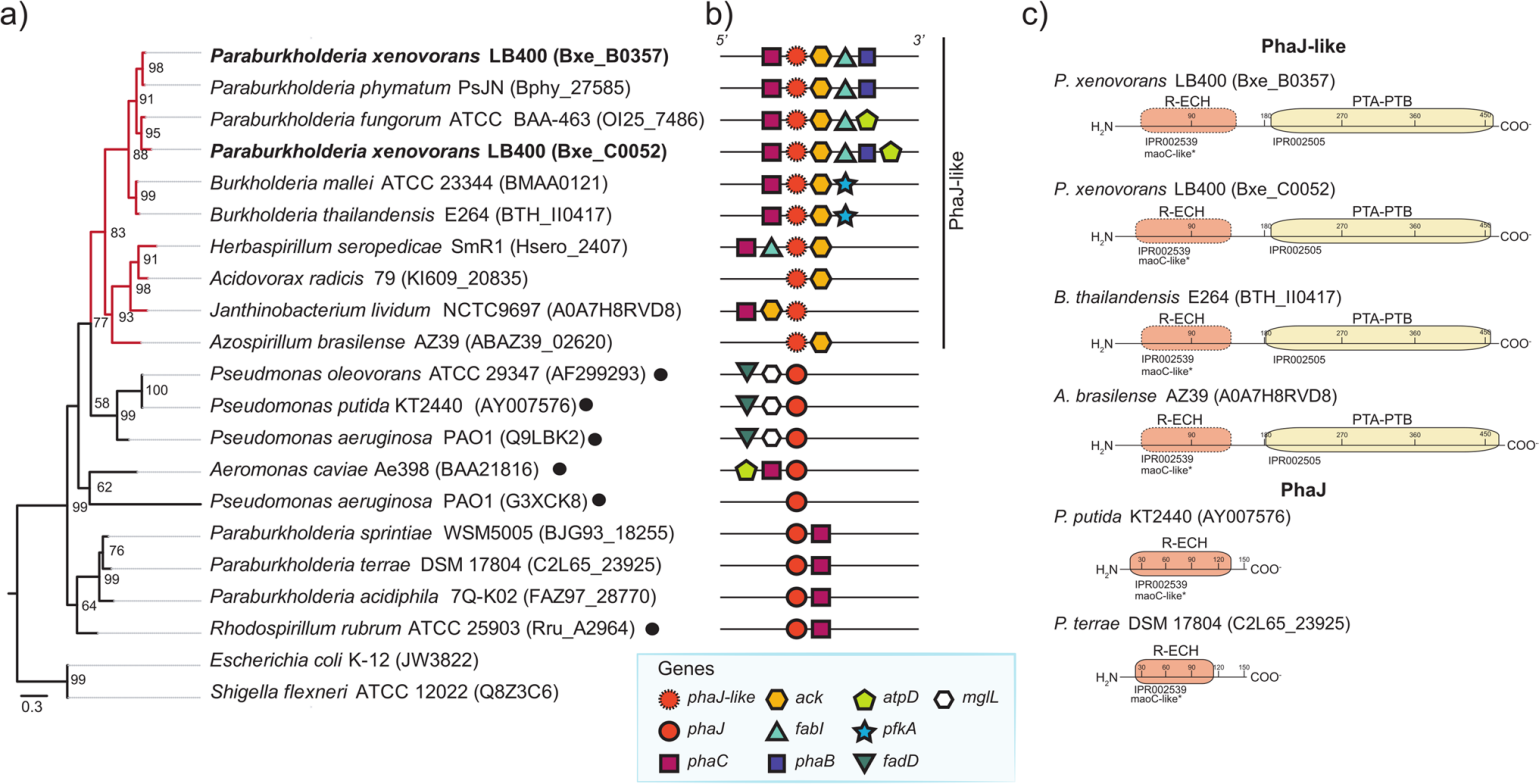
*P. xenovorans* LB400 possesses two potential *R*-specific enoyl-CoA hydratases (PhaJ-like) harboring a phosphate acetyl-butyryl transferase (PTA-PTB) domain. **a** Bayesian phylogeny of the *R*-specific hydratase domain (*R*-ECH) of the PhaJ-related sequences found in strain LB400 and other reference Gammaproteobacteria. Black dots show protein sequences of functionally characterized enzymes in PHA synthesis. The yellow clade corresponds to sequences with the PTA-PTB domain. The enzymes JW3822 and Q8Z3C6 of the *β*-oxidation pathway from Enterobacteriaceae were used as an outgroup. **b** Genomic context of *phaJ* and *phaJ*-like genes in selected strains. Unidentified genes are not included. **c** Structural domains predicted through the InterProScan tool (Paysan-Lafosse et al. 2023)

The *R*-specific enoyl-CoA hydratases exist in diverse Proteobacteria and are interesting metabolic targets for tailoring PHA monomer composition (Jung et al. 2019). The PTA-PTB domain from these PhaJ-like enzymes was first reported by Álvarez-Santullano et al. (2021) in a genome-wide metabolic analysis including representative strains of the *Burkholderia *sensu lato group (i.e., *Burkholderia*, *Paraburkholderia*, *Trinickia*, *Caballeronia*, *Mycetohabitans*, and *Robbsia* genera). Phosphate acetyl-butyryl transferase enzymes (Pta) commonly interconvert acetyl-CoA or butyryl-CoA into acetyl-phosphate or butyryl-phosphate, respectively; although, a non-catalytic PTA-PTB domain has been shown to play a regulatory role in a bifunctional malic enzyme (Wolfe 2005; Bologna et al. 2007). The PhaJ enzymes are important for PHA synthesis from related carbon sources of different chain lengths (Tsuge et al. 2015). Therefore, the *R*-ECH domain of the PhaJ-like enzymes of strain LB400 was phylogenetically analyzed, including reference *R*-ECH sequences able to synthesize PHA_scl_ and PHA_mcl_ precursors (Fig. 6). The PhaJ-like enzymes of strain LB400 were arranged according to genus affiliation and formed a separate clade from the reference PhaJ enzymes, lacking the PTA-PTB domain (Fig. 6A). The PhaJ-like enzymes of strain LB400 are phylogenetically closer to the PhaJ2 of *Pseudomonas aeruginosa* PAO1 that yields PHA_mcl_ precursors of C6–C10 chain lengths (Tsuge et al. 2015). Whereas PhaJ enzymes specific for C4 to C6 precursors are arranged in a more distant clade (Hisano et al. 2003; Reiser et al. 2000). The PhaJ-like enzymes of strain LB400 and other *Paraburkholderia* are conserved and are located next to genes encoding a PHA synthase (*phaC*), acetate kinase (*ackA*), ketoacyl-ACP reductase (*fabI*), and *R*−3HB-CoA reductase (*phaB2*) (Fig. 6). Otherwise, *Burkholderia* species encode a phosphofructokinase enzyme (PfkA) that replenishes the glycolytic EMP pathway (Fig. 6B).

An additional pathway to synthesize *R-*3HV-CoA is the epimerization of *S-*3HV-CoA by the multi-functional enzyme 2-*trans*-enoyl-CoA hydratase (ECH)/(*S*)-3-hydroxyacyl-CoA (*S-*3HA-CoA) dehydrogenase (HADH) (Steinbüchel 2001). This PHA synthetic pathway is scarcely reported in the literature, despite being a relevant target to tailor PHA monomer composition or to increase the PHA productivity from related carbon sources. *P. xenovorans* LB400 harbors an ECH/HADH (BxeC0280) with 37% of total amino acid sequence identity with the FadJ enzyme of *E. coli* K12, which is expressed under anaerobic conditions and performs ECH, 3HADH, and *S-*3HA-CoA epimerase activities (Yang et al. 1988; Cho et al. 2006).

**Fig. 7:**
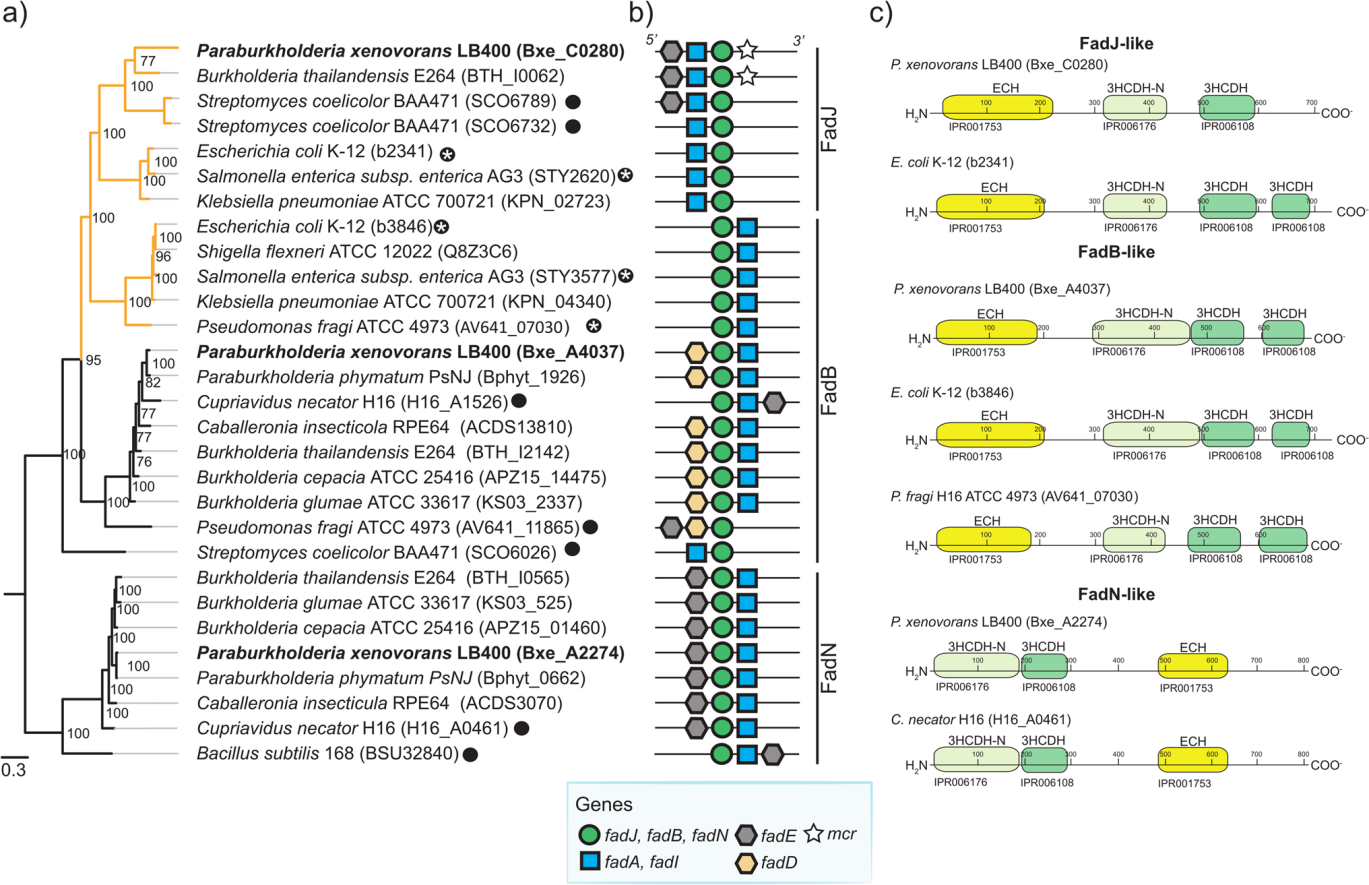
*P. xenovorans* LB400 possesses a FadJ/B enzyme with potential (*S*)−3-hydroxyacyl-CoA epimerase function. **a** Bayesian phylogeny of the enoyl-CoA hydratase/(*S*)−3-hydroxyacyl-CoA dehydrogenase (ECH/HADH) enzymes FadJ, FadB, and FadN from strain LB400 and reference strains. Black dots show protein sequences related to functionally characterized ECH/HADH enzymes, while white asterisks indicate sequences with epimerase function. The yellow clade corresponds to sequences proposed as probable epimerases. **b** Genomic context of *fadJ, fadB*, and *fadN* genes identified in selected strains (unidentified genes are not included). **c** Structural domains predicted with the InterProScan tool (Paysan-Lafosse et al. 2023)

The hypothesized *S-*3HA-CoA epimerase function of the *fadJ* and *fadB* genes of *P. xenovorans* LB400 was assessed through phylogenetic analysis, gene context, and domain architecture. Protein gene sequences were compared to FadJ enzymes previously characterized with epimerase function (Fig. 7). The FadJ amino acid sequence of strain LB400 is phylogenetically related to the FadJ and FadB enzymes from *E. coli* K12, *Salmonella enterica* AG3, and *Pseudomonas fragi* ATCC 4973 that exhibit epimerase activity (Fig. 7A). Additionally, the *fadJ* gene of strain LB400 shows similar arrangements with those of *E. coli* (e.g.,* fadAJ*), while the other genes present inverted arrangements (e.g.,* fadBA*, *fadNA*). This FadJ shows an *Escherichia*-like domain architecture (Volodina & Steinbüchel 2014). *P. xenovorans* LB400 has two additional multifunctional ECH/HADH enzymes. The FadB (BxeA2274) amino acid sequence presents an *Escherichia*-like topology and is similar to the FadB of *P. fragi* ATCC4973 that performs ECH, HADH, and epimerase activities during the *β*-oxidation of fatty acids under aerobic and anaerobic conditions (Imamura et al. 1990). The third ECH/HADH encoded by the BxeA4037 gene is grouped in a clade within Burkholderiaceae strains without epimerase function and shows a *Burkholderia-*like domain architecture (Volodina & Steinbüchel 2014). However, the closest relative is the FadN that oxidizes linear and branched fatty acids in *Bacillus subtilis* 168 under aerobic and anaerobic conditions (Matsuoka et al. 2007).

Another *R-*3HV-CoA synthetic pathway consists of the reduction of 3-ketovaleryl-CoA by a 3-ketovaleryl-ACP reductase (FabG) or the PhaB enzyme with the concomitant oxidation of NADPH (Fig. 5). Finally, *P. xenovorans* LB400 harbors the gene encoding the 3-ketothiolase enzyme (BktB), for propionyl-CoA condensation with acetyl-CoA into 3-ketovaleryl-CoA, which can be funneled into PHA synthesis. The BktB encoding gene (BxeA2335) is located 4.8 kb downstream of the *phaC1AB1R* gene cluster.

### Assessment of *P. xenovorans* LB400 as a biocatalyst for scaling-up fermentation processes

**Fig. 8:**
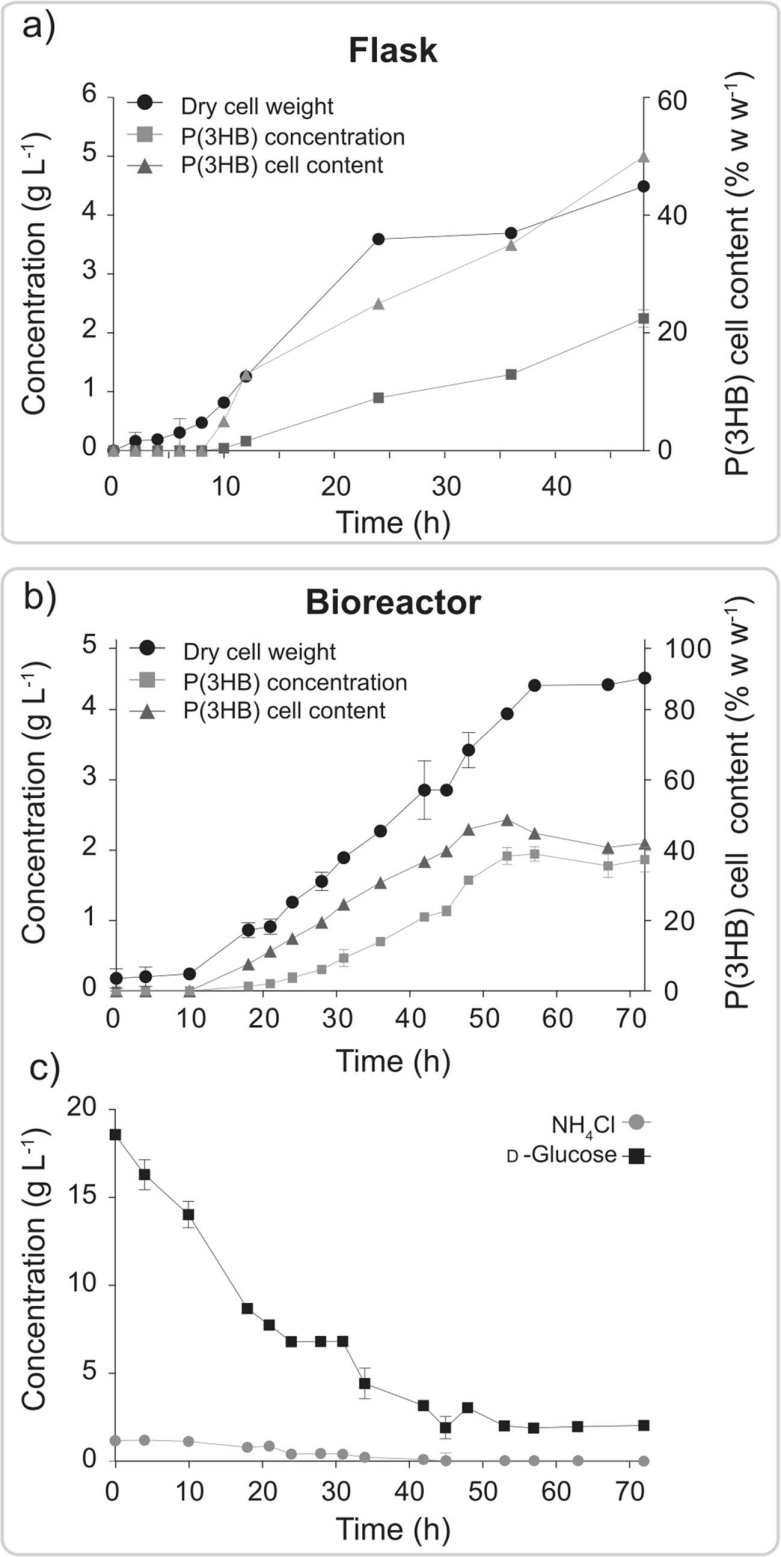
Synthesis of P(3HB) by *P. xenovorans* LB400 with d-glucose (20 g L^−1^) in flasks and a 2.5-L bioreactor. **a** P(3HB) synthesis during growth of strain LB400 in agitated flask cultures of 50 mL. **b***, ***c** Growth and P(3HB) synthesis (**b**) and substrate consumption (**c**) by strain LB400 in a bioreactor with a 2.5-L working volume

The metabolic versatility displayed by *P. xenovorans* LB400 represents an opportunity to develop bioprocesses to synthesize different PHA polymers. Therefore, the scale-up of P(3HB) synthesis by strain LB400 was evaluated from flask cultures up to a 2.5-L bioreactor. Flask cultures of strain LB400 using 20 g L^−1^ of d-glucose reached up to 4.5 g L^−1^ of DCW with a P(3HB) content of 50% w w^−1^, obtaining a final polymer concentration of ~ 2.2 g L^−1^ (Fig. 8a). These cultures reached the exponential and stationary phases at 5 and 24 h incubation, respectively, while P(3HB) production began at 10 h and increased until 48 h incubation. In the batch bioreactor, *P. xenovorans* LB400 reached 4.2 g L^−1^ of DCW with a P(3HB) content of 48.70% w w^−1^ after 53 h of incubation (Fig. 8b). The maximum P(3HB) concentration achieved by strain LB400 was 2.12 g L^−1^; although, a decrease in the polymer content was observed during the stationary phase, reaching 1.92 g L^−1^. Culture started with a 100% dissolved oxygen saturation, decreasing to 15–20% during the exponential growth phase. The P(3HB) yield using d-glucose as substrate (*Y*_*p/s*_) was 0.096 g P(3HB) g^−1^ glucose. Strain LB400 had a lag phase of 10 h in the reactor and reached the stationary phase at ~ 55 h of incubation.

## Discussion

This study highlights the potential of *P. xenovorans* LB400 as a biocatalyst for producing PHA polymers, specifically P(3HB) and P(3HB-*co-*3HV), from different sugars, or in combination with valerate in fed-batch cultures. Strain LB400 synthesized P(3HB) from the sugars d-glucose, d-mannitol, d-gluconate, and d-xylose. Biomass and P(3HB) production were observed with DCW values ranging from 1.00 to 1.39 g L^−1^ and polymer contents between 21 and 43% (w w^−1^). The highest intracellular P(3HB) content (43%) was observed in d-mannitol-grown cells. P(3HB) synthesis from d-mannitol has been scarcely studied. d-Mannitol is a major sugar alcohol in seaweed with a high content of carbon atoms in a reduced redox state (Xiao et al. 2024). Seaweed has been used for the synthesis of PHAs due to its high sugar content and absence of lignin (Tuma et al. 2020). Strain LB400 showed interesting production levels compared to previous studies. The marine strain *Burkholderia* sp. AIU M5M02 synthesizes P(3HB) from d-fructose, and to a lesser extent, with d-mannitol*,* d-glucose, and d-xylose, reaching 40% P(3HB) of DCW using 10 g L^−1^ d-mannitol after 24 h of growth (Yamada et al. 2018). On the other hand, the marine Gammaproteobacterium *Cobetia amphilecti* reached a DCW of 4.2 g L^−1^ with a P(3HB) content of 76% w w^−1^ when grown on 20 g L^−1^ d-mannitol after 4 days (Gnaim et al. 2022). These results place strain LB400 as a suitable biocatalyst for PHA production using the analyzed carbon sources that are relevant sugars for biotechnology, such as those derived from algal (d-mannitol) and lignocellulosic (d-xylose) feedstocks. Moreover, using LB400 metabolic capabilities for waste valorization may be an advantage to minimize costs for PHA production. For instance, seaweed wastes from phycocolloid extraction industries are sugar-rich substrates that are mostly underexploited (Tuma et al. 2020).

*P. xenovorans* LB400 was able to synthesize the P(3HB-*co-*3HV) copolymer by adding a pulse of valerate to cultures growing on d-glucose, d-mannitol, d-gluconate, or d-xylose. The addition of 1 g L^−1^ valerate increased the DCW values from 1.79 to 2.29 g L^−1^ on average and led to the synthesis of P(3HB-*co-*3HV) copolymer with varying 3HV monomer compositions (28–43 mol%). d-Xylose combined with valerate yielded the highest 3HV incorporation (43%). The use of structurally diverse sugars for tuning P(3HB-*co-*3HV) monomer composition has been scarcely studied. The ability of strain LB400 to synthesize P(3HB-*co*−3HV) with adjustable monomer ratios from different sugars will allow tailoring polymer properties with a focus on specific industry requirements by establishing a suitable bioprocess design. Although the sugar selected for cultivating *P. xenovorans* LB400 influenced the 3HB:3HV monomer ratio, the co-feeding of structurally diverse fatty acids may also influence the incorporation of 3HV monomers (Han et al. 2013; Kiun et al. 2019). In the present study, the P(3HB*-co*−3HV) produced by *P. xenovorans* LB400 showed similar monomer compositions compared to that of *Alcaligenes* sp. SH-69 (24 mol% 3HV) grown on 10 g L^−1^ glucose and 1 g L^−1^ valerate and *C. necator* H16 (21 mol% 3HV) grown on 20 g L^−1^ d-glucose and 5 g L^−1^ levulinate (Choi et al. 2003; Wang et al. 2013). *Burkholderia* sp. IS-01 reached 47–71 mol% 3HV when grown with 5 g L^−1^ valerate or 5 g L^−1^ levulinate plus 20 g L^−1^ gluconate (Kim et al. 2009). *Paraburkholderia sacchari* LMG 19450 and *Alcaligenes* sp. SH-69 showed 6–18 mol% 3HV when grown with 5–10 g L^−1^ d-glucose and 1 g L^−1^ propionate (Silva et al. 2000; Choi et al. 2003). Higher 3HV incorporation is expected from valerate or levulinate as the *R-*3HV-CoA synthetic pathways from fatty acid *β*-oxidation intermediates possess fewer catalytic steps and are more redundant than those from propionate (Fig. 5). However, valerate may directly increase 3HB monomer synthesis as this fatty acid undergoes *β*-oxidation, yielding acetyl-CoA, which is transformed into* R*−3HB-CoA. Accordingly, the mass fraction of 3HB monomers in P(3HB-*co*−3HV) obtained from the cultures fed with sugars plus valerate was 28.80–57.56% higher compared to the P(3HB) mass obtained from all the cultures tested with sugars alone, without valerate. This suggests that valerate addition contributed to 3HB synthesis. The increment of 3HB monomer synthesis upon the addition of valerate could be modulated with suitable culture conditions to customize the 3HB:3HV ratio according to specific applications.

This study showed that sugar types strongly affected biomass production and polymer synthesis by *P. xenovorans* LB400. Genomic analyses provided a comprehensive view of the metabolism of strain LB400, unveiling key molecules and enzymes for PHA synthesis toward tailored monomer compositions. In strain LB400, each sugar is channeled through distinct routes of central carbon metabolism, differing in pathway length and metabolite generation, such as NADPH, which in turn may influence PHA accumulation (Table 1). NADPH is an electron donor of the PhaB reductase, which is a limiting catalytic step in the P(3HB) synthetic pathway of *P. sacchari* LMG 19450 (Morris et al. 2024). In *P. xenovorans* LB400, d-glucose and d-mannitol are metabolized through the OxPP and ED pathways, leading to the generation of 1–3 equivalents of NADPH, a higher number of cofactor molecules than the pathways of the other sugars, resulting in a 12–22% increase in P(3HB) accumulation relative to other sugars. In contrast, D-gluconate is metabolized exclusively through the ED pathway, resulting in reduced NADPH generation and lower P(3HB) accumulation (21.1% w w^−1^) (Table 1). However, d-gluconate supported higher biomass production, likely due to the ED pathway, which requires minimal protein expenses for the efficient synthesis of acetyl-CoA (Table 1) (Nikel et al. 2015). Efficient acetyl-CoA synthesis from d-glucose and d-gluconate in strain LB400 correlates with the increased incorporation of 3HB relative to the 3HV monomers, observed in the P(3HB-*co*−3HV) copolymer.

**Table 1:** Features of sugar metabolic pathways of *P. xenovorans* LB400 for P(3HB) synthesis

Sugar	No. reactions^a^	NADPH^b^	NADH/FADH_2_	ATP^b^	Metabolic pathway^c^
d-Glucose	6	1–2	2.6-3/0	1	PP, ED, EMP
d-Mannitol	8	2–3	2.6-3/0	1	EMP, PP, ED
d-Gluconate	3	0–1	2.6-3/0	1	ED, PP, EMP
d-Xylose	11–13	1–2	3-4/1	1^d^	Weimberg, Krebs cycle
d-Xylose	12	0.7	2.6/0	1	XI, PP, EMP, ED^e^

^a^Number of reactions for the conversion of each sugar to acetyl-CoA

^b^Numbers are 1 mol equivalent

^c^The stoichiometry is constrained by exclusive ED glycolysis in strain LB400 as EMP pathway is incomplete due to the absence of phosphofructokinase enzyme (Álvarez-Santullano et al. 2021)

^d^D-Xylose metabolization through Krebs cycle yields GTP that can be converted into ATP

^e^Metabolization of F6P intermediate via ED pathway was assumed (Nikel et al. 2015)

PP pentose-phosphate, ED Entner-Doudoroff, EMP Embden-Meyerhof-Parnas (lower), XI xylose isomerase, Non-OxPP non-oxidative branch of the PP pathway

Carbon flux partition among pathways is not considered in the analysis

D-Xylose resulted in reduced biomass yield and lower P(3HB) accumulation in *P. xenovorans* LB400 compared to d-glucose and d-mannitol. d-Xylose may be metabolized in strain LB400 through the Weimberg pathway (Tai et al. 2016) or the xylose isomerase pathway. In contrast, *P. sacchari* LMG 19450 and *Burkholderia cepacia* JC-1 only possess the xylose isomerase pathway to metabolize d-xylose, achieving higher biomass production (24–34%) on d-xylose compared to d-glucose, although with reduced P(3HB) accumulation (13–42%) (Cesário et al. 2014; Guamán et al. 2018; Chin et al. 2022). In *P. xenovorans* LB400, the Weimberg pathway may play a significant role in d-xylose catabolism (Tai et al. 2016). This pathway oxidizes d-xylose into the Krebs cycle intermediate, 3-ketoglutarate, involving multiple enzymatic steps for 3HB monomer synthesis, thereby limiting P(3HB) production despite yielding 1–2 equivalents of NADPH (Fig. 4). Furthermore, an increase in Krebs cycle activity antagonizes the P(3HB) synthetic pathway, as the release of coenzyme A inhibits allosterically the PhaA enzyme (Mitra et al. 2022). Notably, d-xylose-grown LB400 cells supplied with valerate exhibited the highest 3HV:3HB ratio of the P(3HB-*co*−3HV) copolymer (43%), which correlates with a lower 3HB synthesis compared to the other sugars. Alternative pathways for 3HV synthesis from d-xylose in strain LB400 cannot be discarded. Propionyl-CoA and acetyl-CoA can be condensed by the BktB 3-ketothiolase into 3-ketovalerate-CoA to be used for 3HV monomer synthesis (Fig. 5). Propionyl-CoA may be produced from Krebs cycle intermediates via the methyl malonyl pathway or the metabolization of amino acids (Steinbüchel & Lütke-Eversloh 2003; Han et al. 2013), which has been used for adjusting 3HV:3HB ratio in P(3HB-*co-*3HV) produced by recombinant *E. coli* cells from non-related sugars (Srirangan et al. 2016).

Genome-guided analyses of the metabolic pathways *P. xenovorans* LB400 revealed further information on key enzymes encoded in its genome, showing interesting features for tuning monomer composition or for increasing polymer yields. *P. xenovorans* LB400 possesses two *R*-specific enoyl-CoA hydratases for synthesizing *R*−3HV-CoA, and both harbor a PTA-PTB domain (Fig. 6) (Álvarez-Santullano et al. 2021). The PhaJ-like enzymes from strain LB400 phylogenetically clustered with C4–C8 utilizing enzymes, suggesting specificity towards PHA_scl_ or PHA_mcl_ precursors (Fig. 6) (Tsuge et al. 2015). Additionally, according to phylogenetic analyses, *P. xenovorans* LB400 possesses the FadJ and FadB enzymes that may epimerize *S-*3HA-CoA into the synthetic precursor of PHA monomers (Yang et al. 1988; Imamura et al. 1990). Several studies have attempted to produce PHAs via epimerization of *S*−3HA-CoA in wild-type strains (Steinbüchel et al*.,* 2001; Fiedler et al. 2002), which were unsuccessful unless mutations on the enzymes involved in fatty acid *β*-oxidation were conducted (Olivera et al. 2001; Qi et al. 1998). Variations in the ECH/HADH enzymes FadJ, FadB, and FadN associated with their activity (e.g., epimerase activity), substrate specificity, and oxygen conditions demonstrate the metabolic versatility of strain LB400 during fatty acid *β*-oxidation. These enzymes can be further studied for the channeling of *β*-oxidation intermediates toward PHA synthesis.

Due to the metabolic versatility and robustness of *P. xenovorans* LB400 for P(3HB) and P(3HB-*co*−3HV) synthesis, its potential for scaling up PHA production from d-glucose was evaluated in both flask and bioreactor cultures. After doubling the d-glucose concentration in flask cultures, *P. xenovorans* LB400 increased biomass production and P(3HB) accumulation by 75% and 80%, respectively, reaching a polymer concentration of 2.1 g L^−1^. Interestingly, *P. sacchari* LMG 19450 showed the same trend by doubling the d-glucose concentration in flask cultures (Cesário et al. 2014). *P. xenovorans* LB400 maintained the P(3HB) concentration in bioreactor culture, reaching a polymer concentration of 1.9 g L^−1^ and productivity of 0.04 g L^−1^ h^−1^. Lower biomass and P(3HB) production are reported in *Rhodococcus pyridinivorans* BRST1-1, reaching a polymer concentration of 1.4 g L^−1^ and productivity of 0.02 g L^−1^ h^−1^ in a 6-L batch bioreactor using 34 g L^−1^ D-fructose (Trakunjae et al. 2021). Higher productivities have been reported in 3–7-L batch bioreactors using different bacterial strains. *C. necator* H16, *Iodobacter* sp. PCH 194, and *Halomonas bluephagenesis* TD01 achieved between 15 and 32 g DCW L^−1^, with P(3HB) concentrations ranging from 11 to 24 g L^−1^ and productivities of 0.042–0.54 g L^−1^ h^−1^ using d-fructose, d-glucose plus tryptone, and d-glucose as carbon sources, respectively (Ma et al. 2020; Kumar et al. 2021; Nygaard et al. 2021). P(3HB) production by *P. xenovorans* LB400 may be improved by adjusting agitation and aeration strategies, which may generate shear stress. A suitable bioreactor design toward high-density cultures can be employed, as shown with *P. sacchari* LMG 19450 in an air-lift fed-batch bioreactor (da Cruz-Pradella et al. 2010). Strategies can be employed to mitigate shear stress and support high-cell-density cultivation to achieve higher PHA yields. *P. xenovorans* LB400 harbors a diverse repertoire of stress response mechanisms when exposed to various environmental factors, including nutrient limitation, high salinity, and exposure to toxic and oxidizing compounds (Méndez et al. 2025). These mechanisms provide strain LB400 with robustness for scaling up and optimizing PHA production under the oxidative and mechanical stress that entail high-cell-density cultures and the utilization of different carbon sources.

## Conclusions

The PCB- and aromatic-degrading model bacterium *P. xenovorans* LB400 synthesized the P(3HB) homopolymer and the P(3HB-*co-*3HV) copolymer from various sugars and valerate. The metabolic flexibility observed in *P. xenovorans* LB400 represents an advantage for the potential utilization of diverse carbon sources found in low-cost and more sustainable feedstocks, within the NGIB scope, including lignocellulosic materials and seaweed wastes. The combination of selected sugars with valerate enabled modulation of the P(3HB-*co-*3HV) monomer composition, providing a strategy to synthesize customized polyesters. In silico genomic analyses revealed key metabolic factors influencing bacterial growth, PHA accumulation, and monomer composition, including NADPH generation and capability to synthesize the *R-*3HB-CoA precursor via specific sugar metabolic pathways. The metabolic versatility of *P. xenovorans* LB400 enables the utilization of multiple pathways to synthesize 3HV monomers from fatty acids such as valerate. Metabolic enzymes involved in PHA precursor synthesis in strain LB400 constitute a complex and robust system for producing PHAs from fatty acids. Notably, *P. xenovorans* LB400 successfully produced P(3HB) in a 2.5-L bioreactor, highlighting its potential for scaling up production of tailored PHAs to obtain customized bioplastics for high-value biotechnological applications, including those in the medical field.

## Supplementary Information

Below is the link to the electronic supplementary material.

ESM 1 (DOCX 270 KB)

## Data Availability

Genome data of strain *Paraburkholderia xenovorans* LB400 was retrieved from RefSeq (Genome assembly accession number GCF_000013645.1) and KEGG (accession number T00340) databases. Metabolic reconstruction data is provided in the supplementary file. Reference genome data was retrieved from RefSeq database, and the respective sequence accession number is provided in Fig. 6 and Fig. 7.
