# Supplementary material for: Versatile sugar and valerate metabolic pathways in Paraburkholderia xenovorans LB400 enable tailored poly(3-hydroxybutyrate-co-3-hydroxyvalerate) production
Source: Appl Microbiol Biotechnol. 2025 Oct 29;109:239. doi: 10.1007/s00253-025-13599-8 (PMC12568896; doi:10.1007/s00253-025-13599-8)
Supplement: Supplementary file 1 [file 253_2025_13599_MOESM1_ESM.docx]

*Supplementary material:*

**Versatile sugar and valerate metabolic pathways in *Paraburkholderia xenovorans* LB400 enable tailored poly(3-hydroxybutyrate-*co*-3-hydroxyvalerate) production**

Mario I. Sepúlveda^1,2^, Viviana Urtuvia^1,3^, Natalia Álvarez-Santullano^1^, Pamela Villegas^1,4,5^, Jacqueline Vásquez-Navarrete^1,2^, Valentina Saffirio^1,2^, Alvaro Díaz-Barrera^3^, Myriam González^1^, Jose Gregório C. Gomez^6^, Valentina Méndez^1,2^, and Michael Seeger^1,2,^*

^1^Laboratorio de Microbiología Molecular y Biotecnología Ambiental, Departamento de Química & Centro de Biotecnología Daniel Alkalay Lowitt, Universidad Técnica Federico Santa María, Avenida España 1680, 2390123, Valparaíso, Chile.

^2^Millenium Nucleus Bioproducts, Genomics and Environmental Genomics (BioGEM), Avenida España 1680, 2390123, Valparaíso, Chile.

^3^Escuela de Ingeniería Bioquímica, Pontificia Universidad Católica de Valparaíso, Av. Brasil 2147 Casilla 4059, Valparaíso, Chile

^4^Programa Doctorado en Ciencias de Materiales e Ingeniería de Procesos, Universidad Tecnológica Metropolitana, Santiago 8940000, Chile

^5^Nano-Biotechnology Applied Laboratory, Instituto de Agroindustría, Universidad de La Frontera, Temuco, Chile

^6^Department of Microbiology, Institute of Biomedical Sciences, University de São Paulo, São Paulo, Brazil.

* Correspondence: michael.seeger@usm.cl; michael.seeger@gmail.com

1. **Supplementary Tables**

**Table S1.** FT-IR analysis of functional groups of P(3HB) polymer synthesized by *P. xenovorans* LB400 from *D-*glucose, *D-*mannitol, *D-*gluconate or *D-*xylose

| Polymer | Functional groups | Wave number  (cm^-1^) | Assignation |
| --- | --- | --- | --- |
| P(3HB) | O-C=O | 1720–1727 | PHA |
|  | -CH_3_ | 1379–1380 | P(3HB) |
|  | -CH_2_- | 1453 | P(3HB) |
|  | OH | 3435–3439 | PHA |

Assignation is the polymer type associated with the identified functional group, respectively.

**Table S2.** Chemical shifts from the ^1^H-NMR and ^13^C-NMR spectra of P(3HB) produced by strain LB400 using *D-*glucose, *D-*mannitol, *D-*gluconate, or *D-*xylose.

| Polymer | Atom (No.) | ^1^H chemical shift^a^ (ppm) | Atom (No.) | ^13^C chemical shift^a^ (ppm) |
| --- | --- | --- | --- | --- |
| P(3HB) | --- | --- | b1 | 169.18 |
|  | a2 | 2.57 to 2.45 | b2 | 40.82 |
|  | a3 | 5.25 | b3 | 67.64 |
|  | a4 | 1.25 | b4 | 19.79 |

^a^Solvent CDCL_3_. Chemical shifts are related to the residual signals of the solvent (^1^H, 7.25 ppm; ^13^C, 77.04 ppm). For details see text, Figure S1.

**Table S3.** FT-IR analysis of PHA polymers functional groups synthesized by *P. xenovorans* LB400 from valerate plus *D-*glucose, *D-*mannitol, *D-*gluconate, or *D-*xylose.

| **Polymer** | **Functional groups** | **Wavenumber**  **(cm^-1^)** | **Assignation** |
| --- | --- | --- | --- |
| P(3HB-*co*-3HV) | O-C=O | 1723–1724 | PHA |
|  | -CH_3_ | 1379–1380 | P(3HB) |
|  | -CH_2_- | 1453–1459 | P(3HB) |
|  | OH | 3436–3437 | PHA |

Assignation is the polymer type associated with the identified functional group, respectively.

**Table S4.** Chemical shifts from the ^1^H-NMR and ^13^C-NMR spectra of P(3HB) and P(3HB-*co*-3HV) produced by strain LB400 from valerate plus *D-*glucose, *D-*mannitol, *D-*gluconate, or *D-*xylose.

| **Polymer** | **Atom (No.)** | **^1^H chemical shift^a^ (ppm)** | **Atom (No.)** | **^13^C chemical shift^a^ (ppm)** |
| --- | --- | --- | --- | --- |
| P(3HB-c*o*-3HV) | --- | --- | b1, b9 | 169.36 |
|  | a2, a8 | 2.57 to 2.45 | b2, b8 | 40.82 |
|  | a3, a7 | 5.25 | b3, b7 | 67.70 |
|  | a4 | 1.25 | b4 | 19.80 |
|  | a5 | 1.59 | b5 | 9.39 |
|  | a6 | 1.63 | b6 | 26.8 |

^a^Solvent CDCL_3_. Chemical shifts are related to the residual signals of the solvent (^1^H, 7.25 ppm; ^13^C, 77.04 ppm). For details see text, Figure S2.

**Table S5. Predicted enzymes of sugar metabolism for the synthesis of P(3HB) by *P. xenovorans* LB400**

| **Locus tag** | **Gene product** | **Cofactor** | | **Id. (%)** | | | | | **Cov. (%)** | | **e-val**  **(log_10_)** | | | **Organism** | | | |  |
| --- | --- | --- | --- | --- | --- | --- | --- | --- | --- | --- | --- | --- | --- | --- | --- | --- | --- | --- |
| **Peripheric catabolism of sugars** | |  | |  | | | | |  | |  | | |  | | | |  |
| Bxe_A3454 | Bifunctional glucokinase – HexR (Glk) | ATP | | 44 | | | | | 96 | | -83 | | | *Shigella flexneri* 2a | | | |  |
| Bxe_B0216 | Bifunctional glucokinase (PpgK) | PolyP | | 30 | | | | | 76 | | -22 | | | *Mycobacterium tuberculosis* H37Rv | | | |  |
| Bxe_A0730 | *D*-Mannitol dehydrogenase (MtlK) | NADPH | | 38 | | | | | 77 | | -73 | | | *Cereibacter* *spheroidetes* HR | | | |  |
| Bxe_A4286 | Fructokinase (ScrK) | ATP | | 30 | | | | | 65 | | -24 | | | *E. coli* EC3132 | | | |  |
| Bxe_A0592 | *D*-Gluconate kinase (GntK) | ATP | | 45 | | | | | 95 | | -44 | | | *E. coli* K12 | | | |  |
| **Catabolism of D-xylose via Weimberg pathway** | |  | | |  | | | |  | | |  | | |  | | |  |
| Bxe_C1363 | *D*-xylose dehydrogenase (XylB)^a^ | NADP^+^ | | | - | | | | - | | | - | | | - | | |  |
| Bxe_C1362 | *D-*Xylonolactonase (XylC)^a^ | - | | | - | | | | - | | | - | | | - | | |  |
| Bxe_C1359 | *D-*Xylonate dehydratase (XylD)^a^ | - | | | - | | | | - | | | - | | | - | | |  |
| Bxe_C1358 | 2-Keto-3-deoxyxylonate dehydratase (XylX)^a^ | - | | | - | | | | - | | | - | | | - | | |  |
| Bxe_C1357 | 2,5-Dioxopentanoate dehydrogenase (KivD) | NAD(P)^+^ | | | 44 | | | | 99 | | | -133 | | | *Bacillus subtilis* 3NA | | |  |
| **Catabolism of D-Xylose via isomerase pathway** | |  | | |  | | | |  | | |  | | |  | | |  |
| Bxe_B2622 | D-Xylose isomerase (XylA) | - | | | 67 | | | | 100 | | | 0 | | | *E. coli* K12 | | |  |
| Bxe_A0232 | *D-*Xylulose 5-kinase | - | | | 49 | | | | 98 | | | -144 | | | *E. coli* K12 | | |  |
| **Oxidative branch of the pentose-phosphate (PP) pathway (OxPP)** | | | | | | | | |  | |  | | |  | | | |  |
| Bxe_A3452 | *D-*Glucose-6-phosphate dehydrogenase (Zwf1) | NADP^+^ | | 49 | | | | | 92 | | -154 | | | *Mycobacterium smegmatis* mc2115 | | | |  |
| Bxe_B0215 | *D-*Glucose-6-phosphate dehydrogenase (Zwf2) | NADP^+^ | | 43 | | | | | 73 | | -135 | | | *Anabaena* sp. PCC 7120 | | | |  |
| Bxe_B1764 | *D-*Glucose-6-phosphate dehydrogenase (Zwf3) | NADP^+^ | | 52 | | | | | 92 | | -170 | | | *Sinorhizobium meliloti* 1021 | | | |  |
| Bxe_B0215 | 6-Phosphogluconolactonase (Pgl) | - | | 37 | | | | | 81 | | -37 | | | *P. putida* KT2440 | | | |  |
| Bxe_B1341 | 6-Phosphogluconate dehydrogenase (Pgdh1) | NADP^+^ | | 50 | | | | | 78 | | -113 | | | *Gluconobacter oxydans* 621H | | | |  |
| Bxe_B2210 | 6-Phosphogluconate dehydrogenase (Pgdh2) | NADP^+^ | | 74 | | | | | 100 | | 0 | | | *E. coli* K12 | | | |  |
| **ED pathway** | |  | |  | | | | |  | |  | | |  | | | |  |
| Bxe_A0589 | 6-Phosphogluconate dehydratase (Edd) | - | | 62 | | | | | 98 | | 0 | | | | *P. aeruginosa* PAO1 | | |  |
| Bxe_A0590 | KDPG aldolase (Eda) | - | | 45 | | | | | 100 | | -59 | | | | *Dickeya dadantii* 3937 | | |  |
| **Non-oxidative branch of the PP pathway (NonOxPP)** | | | | | | | | |  | |  | | | |  | | |  |
| Bxe_A0565 | Transketolase (Tkt1) | - | | 66 | | | | | 99 | | 0 | | | | *Vibrio cholerae* ATCC 39315 | | |  |
| Bxe_B2448 | Transketolase (Tkt2) | - | | | 67 | | | | 98 | | | 0 | | | *C. necator* H16 | | |  |
| Bxe_A3318 | Transaldolase (Tal) | - | | | 88 | | | | 100 | | | 0 | | | *S. flexneri* 2a | | |  |
| Bxe_A0463 | Ribulose-5-phosphate 3-epimerase (Rpe1) | - | | | 70 | | | | 98 | | | -114 | | | *S. flexneri* 2a | | |  |
| Bxe_B2446 | Ribulose-5-phosphate 3-epimerase (Rpe2) | - | | | 61 | | | | 98 | | | -96 | | | *S. flexneri* 2a | | |  |
| Bxe_A2538 | Ribose-5-phosphate isomerase (Rpi) | - | | | 66 | | | | 100 | | | 0 | | | *E. coli* K12 | | |  |
| **Upper branch of the Embden-Meyerhof-Parnas (EMP) pathway** | | | | |  | | | |  | | |  | | |  | | |  |
| Bxe_A0691 | Fructose bi-phosphate aldolase (CbbA1) | - | | | | 72 | | 100 | | | | 0 | | | | *Xanthobacter flavus* H4-14 | | |
| Bxe_B1334 | Fructose bi-phosphate aldolase (CbbA2) | - | | | | 56 | | 100 | | | | -126 | | | | *C. necator* H16 | | |
| Bxe_B2447 | Fructose bi-phosphate aldolase (CbbA3) | - | | | | 78 | | 100 | | | | 0 | | | | *C. necator* H16 | | |
| Bxe_A0920 | Fructose 1,6-bisphosphatase (Fbp1) | - | | | | 72 | | 98 | | | | -180 | | | | *P. aeruginosa* PAO1 | | |
| Bxe_B2450 | Fructose 1,6-bisphosphatase (Fbp2) | - | | | | 57 | | 93 | | | | -141 | | | | *C. necator* H16 | | |
| Bxe_A2287 | Glucose-6-phosphate isomerase (Pgi) | - | | | | 57 | | 95 | | | | 0 | | | | *Deinococcus geothermalis* AG3A | | |
| **Lower branch of EMP pathway and tricarboxylic acid cycle** | | | | | | | | | | | | | | | | | |  |
| Bxe_A1699 | PEP synthase, water dikinase (PpsA) | AMP | | | | 64 | | 99 | | | | 0 | | | | *E. coli* K12 | |  |
| Bxe_B2644 | Pyruvate dikinase (PpdK) | ADP | | | | 43 | | 93 | | | | -124 | | | | *Cenarchaeum symbiosum* TA1 | |  |
| Bxe_A0690 | Pyruvate kinase (KpyK) | ADP | | | | 49 | | 98 | | | | -156 | | | | *Buchnera aphidicola* L23 | |  |
| Bxe_A1573 | Enolase (Eno) | - | | | | 91 | | 100 | | | | 0 | | | | *R. pickettii* 12J | |  |
| Bxe_A0613 | Phosphoglycerate mutase (Pgm1) | - | | | | 35 | | 68 | | | | -49 | | | | *P. aeruginosa* PAO1 | |  |
| Bxe_A0689 | Phosphoglycerate kinase (Ppk) | ADP | | | | 86 | | 99 | | | | 0 | | | | *R. solanacearum* GMI1000 | |  |
| Bxe_A0566 | Glyceraldehyde-3-phosphate dehydrogenase | NAD^+^ | | | | 85 | | 100 | | | | 0 | | | | *C. necator* H16 | |  |
| Bxe_A0283 | Malic enzyme (MaeB1) | NADP^+^ | | | | 63 | | 100 | | | | 0 | | | | *E. coli* K12 | |  |
| Bxe_A0557 | Malic enzyme (MaeB2) | NADP^+^ | | | | 61 | | 99 | | | | 0 | | | | *E. coli* K12 | |  |
| Bxe_A3355 | Malic enzyme (MaeB3) | NADP^+^ | | | | 61 | | 99 | | | | 0 | | | | *E. coli* K12 | |  |
| Bxe_B2147 | Oxaloacetate decarboxylase (Oad) | - | | | | 41 | | 97 | | | | -60 | | | | *P. aeruginosa* PAO1 | |  |
| Bxe_A4422 | PEP carboxykinase (PckG) | GTP | | | | 60 | | 98 | | | | 0 | | | | *M. smegmatis* ATCC 700084 | |  |
| Bxe_A3412 | Phosphoenolpyruvate carboxylase (Ppc) | - | | | | 61 | | 95 | | | | 0 | | | | *R. solanacearum* GMI 1000 | |  |
| Bxe_A1541 | Pyruvate dehydrogenase (PdhE) | NAD^+^ | | | | 79 | | 100 | | | | 0 | | | | *C. necator* H16 | |  |
| **Synthesis of PHB** | |  |  | | | |  | | | | |  |  | | | | |  |
| Bxe_A2342 | 3-ketothiolase (PhaA)^b^ | - | | | | - | | | | - | | - | | | | | - |  |
| Bxe_A2341 | 3-ketobutyryl-CoA dehydrogenase (PhaB1)^b^ | NADPH | | | | - | | | | - | | - | | | | | - |  |
| Bxe_B0354 | 3-ketobutyryl-CoA dehydrogenase (PhaB2) | NADPH | | | | 56 | | | | 71 | | -91 | | | | | *Shinorhizobium meliloti* 1021 |  |
| Bxe_A2343 | Polyhydroxyalkanoate synthase (PhaC1)^b^ | - | | | | - | | | | - | | - | | | | | - |  |
| Bxe_B0358 | Polyhydroxyalkanoate synthase (PhaC2) | - | | | | 41 | | | | 98 | | -131 | | | | | *Azorhizobium caulinodans* OS 571 |  |
| Bxe_C0053 | Polyhydroxyalkanoate synthase (PhaC3) | - | | | | 41 | | | | 97 | | -123 | | | | | *Methylobacterium extorquens* IBT 6 |  |

^a^, Functionally characterized by Tai et al., 2016. ^b^, functionally characterized by Urtuvia et al (2019). KDPG, 2-keto-3-deoxy-6-phosphogluconate. PEP, phosphoenolpyruvate. PHA: polyhydroxyalkanoate.

**Table S6. Predicted enzymes for PHA synthesis from fatty acids in *P. xenovorans* LB400**

| **Locus tag** | **Gene product** | **Cofactor** | **Id. (%)** | | **Cov. (%)** | | **e-val**  **(log_10_)** | | **Organism** |
| --- | --- | --- | --- | --- | --- | --- | --- | --- | --- |
| **β-Oxidation of fatty acids** | |  |  | |  | |  | |  |
| Bxe_A2778 | Fatty acid – CoA ligase (FadD) | - | 55 | | 89 | | 0 | | *E. coli* EHEC |
| Bxe_A2774 | Acyl-CoA dehydrogenase (FadE) | FAD | 52 | | 99 | | 0 | | *E. coli* W3110 |
| Bxe_C0280 | (*S*)-3-Hydroxyacyl-CoA dehydrogenase, enoyl-CoA hydratase, epimerase (FadJ) | NAD^+^ | 37 | | 99 | | -124 | | *E. coli* K12 |
| Bxe_A2274 | Long-chain (*S*)-3-hydroxyacyl-CoA dehydrogenase, enoyl-CoA epimerase (FadB) | NAD^+^ | 34 | | 96 | | -115 | | *Pseudomonas fragi* ATCC 4973 |
| Bxe_A4037 | Long-chain (*S*)-3-hydroxyacyl-CoA dehydrogenase (FadN) | NAD^+^ | 39 | | 92 | | -161 | | *Bacillus subtilis* 168 |
| Bxe_A2274 | Fatty acid – CoA ligase (FadD) | - | 43 | | 89 | | -133 | | *E. coli* K12 |
| Bxe_C0281 | 3-ketoacyl-CoA thiolase (FadA) | - | 40 | | 91 | | -79 | | *Aliivibrio salmonicida* LFI1238 |
| Bxe_A2276 | 3-ketoacyl-CoA thiolase (FadA) | - | 56 | | 98 | | 0 | | *E. coli* K12 |
| Bxe_A2777 | 3-ketoacyl-CoA thiolase (FadI) | - | 44 | | 96 | | -98 | | *Idiomarina loihiensis* L2TR |
| **Synthesis of PHAs** | |  | |  | |  | |  |  |
| Bxe_B0357 | *R*-specific enoyl-CoA hydratase – phosphate acetyl-butyryl transferase (PhaJ-like) | - | | 49 (43) | | 30 (61) | | -35  (-66) | *Rhodospirillum rubrum* S1  (*Thermotoga maritima* MSB8) |
| Bxe_C0052 | *R* -specific enoyl-CoA hydratase – phosphate acetyl-butyryl transferase (PhaJ-like) | - | | 49 (56) | | 31 (64) | | -32  (-103) | *R. rubrum* S1  (*S. meliloti* 1021) |
| Bxe_A2776 | *R* -specific enoyl-CoA hydratase (MaoC) | - | | 32 | | 99 | | -29 | *M. tuberculosis* H37Rv |
| Bxe_A2335 | 3-ketothiolase (BktB) | - | | 79 | | 99 | | 0 | *C. necator* H16 |
| Bxe_A1074 | 3-ketoacyl-ACP reductase (FabG) | NADPH | | 66 | | 98 | | -105 | *P. aeruginosa* PAO1 |

Aa, aminoacidic residues. PHAs, polyhydroxyalkanoates. Id, amino acidic sequence identity. Cov, sequence alignment coverage. e-val, e-value. Value in parenthesis indicates alignment parameters of the phosphate acetyl-butyryl transferase domain of PhaJ-like enzymes.

2. **Supplementary Figure**

**Figure S1. Structural characterization of poly(3-hydroxybutyrate) polymer synthesized by *P. xenovorans* LB400 from sugars.** a) Gas chromatography profile of the propylester of 3HB showed a retention time of 8.48 min. The internal standard benzoate showed a retention time of 12.07 min. b) The mass spectrum of 3HB propylester shows the characteristic fragment ions [M-CH_3_]^+^ (*m/z* 131) and [M-CH_3_(CH_2_)_2_O]^+^ (*m/z* 87). c) ^1^H-NMR and d) ^13^C-NMR spectra from P(3HB) produced from mannitol by *P. xenovorans* LB400. The resonance signals were labelled with the appropriate hydrogen and carbon atoms of the 3HB monomer.
